# The hepatic and skeletal muscle ovine metabolomes as affected by weight loss: a study in three sheep breeds using NMR-metabolomics

**DOI:** 10.1038/srep39120

**Published:** 2016-12-14

**Authors:** Mariana Palma, Tim Scanlon, Tanya Kilminster, John Milton, Chris Oldham, Johan Greeff, Manolis Matzapetakis, André M. Almeida

**Affiliations:** 1 ITQB NOVA, Instituto de Tecnologia Química e Biológica António Xavier, Universidade Nova de Lisboa, Oeiras, Portugal; 2 DAFWA – Department of Agriculture and Food Western Australia, Perth, WA, Australia; 3 UWA – University of Western Australia, Perth, WA, Australia; 4 IBET – Instituto de Biologia Experimental e Tecnológica, Oeiras, Portugal; 5 RUSVM – Ross University School of Veterinary Medicine, Basseterre, St. Kitts and Nevis

## Abstract

Sheep are a valuable resource for meat and wool production. During the dry summer, pastures are scarce and animals face Seasonal Weight Loss (SWL), which decreases production yields. The study of breeds tolerant to SWL is important to understand the physiological mechanisms of tolerance to nutritional scarcity, and define breeding strategies. Merino, Damara and Dorper sheep breeds have been described as having different levels of tolerance to SWL. In this work, we assess their liver and muscle metabolomes, and compare the responses to feed restriction. Ram lambs from each breed were divided into growth and feed restricted groups, over 42 days. Tissue metabolomes were assessed by ^1^H-NMR. The Dorper restricted group showed few changes in both tissues, suggesting higher tolerance to nutritional scarcity. The Merinos exhibited more differences between treatment groups. Major differences were related to fat and protein mobilization, and antioxidant activity. Between the Damara groups, the main differences were observed in amino acid composition in muscle and in energy-related pathways in the liver. Integration of present results and previous data on the same animals support the hypothesis that, Dorper and Damara breeds are more tolerant to SWL conditions and thus, more suitable breeds for harsh environmental conditions.

Small ruminants like sheep and goats are particularly important in the tropics and the Mediterranean regions, and are a major source of income and food in small-scale subsistence farming systems. Additionally, in some countries in the Southern hemisphere, such as Australia, New Zealand, Argentina or South Africa, sheep production, particularly for wool, is one of the major commercial products, historically playing an important role in such economies^1^,^2^.

Animal production in the tropics and the Mediterranean is strongly affected by pasture scarcity and quality during the dry summer and autumn months, leading to Seasonal Weight Loss (SWL), as we have demonstrated in South Africa^3^,^4^, Western Africa^5^,^6^, Western Australia^7^ and the Canary Islands^8^,^9^. To counter the effects of SWL, farmers use supplementation to balance the nutritional needs of the animals. Supplementation is expensive and difficult to implement in extensive production systems in developing countries or remote locations^8^. An alternative method for addressing the effects of SWL is the use of sheep breeds that are naturally adapted to this constraint and are able to thrive and more effectively produce in such difficult environments. Understanding the biochemical and physiological mechanisms by which such breeds are able to cope with SWL is therefore very important.

The Australian Merino breed is the basis of the wool, ovine meat, and live animal export markets for that country. The Merino has a long history in Australia after being introduced in 1797^2^, but in the last two decades, market changes and animal welfare policies have led to an increased interest for breeds with other characteristics, particularly regarding the absence of wool (shedding hair sheep), heat tolerance and a natural adaptation to SWL^7^. Recently, two breeds from South Africa, the Damara and the Dorper, have been introduced to Australia. The Damara is a large fat-tailed, hair sheep breed, native to the fringes of the Kalahari Desert in Namibia and South Africa. This breed is well adapted to arid climatic conditions and water scarcity^2^. The Dorper is also a hair sheep breed native to Southern Africa. It was selected by combining the hardiness of the Blackhead Persian indigenous breed with the carcass and meat traits of British Dorset Horn breed^2^. These sheep breeds have been reviewed by Almeida *et al*.^2^.

In a previous work, our team conducted a productive characterization of these breeds and their reaction to SWL. We have studied SWL effect on live weight^10^, carcass and meat characteristics^7^,^11^, in gene expression of regulatory enzymes in the liver^12^, and more recently on the skeletal muscle proteome^13^, and fatty acid composition of muscle^14^ and the Damara fat tail adipose tissue^15^. However, no broad characterization of the muscle and liver metabolomes of these three breeds has ever been conducted.

Recent studies, by our team used Nuclear Magnetic Resonance (NMR) to characterize the mammary gland and milk metabolome of goats under SWL^16^. The study, demonstrated the potential of this approach to help create a more systematic metabolome analysis^16^. Other recent studies in farm animals confirm the potential of NMR technique to metabolomics approaches^17^,^18^,^19^,^20^.

The aim of this work was to characterize the metabolome of the muscle and liver of Merino, Damara and Dorper sheep breeds, and study the effect of feed restriction in these tissues, which are important from the productive and metabolic perspectives. We used an NMR-metabolomics based approach, which, to the best of our knowledge was for the first time applied to these breeds. The results will be of importance to understand which biochemical pathways are associated with SWL tolerance in sheep and that may benefit breeding programs.

## Material and Methods

### Animal experiment

The trial was carried out at the Merredin Research Station in Western Australia, following the experimental design and nutritional treatments previously described^10^. Briefly, a total of 72 six-month-old ram lambs from each of the Merino, Dorper and Damara breeds were divided into the experimental diet groups (12 animals per group: Merino growth, Merino restricted, Dorper growth, Dorper restricted, Damara growth and Damara restricted). All animals were fed on commercial pellets and had free access to drinking water as described^10^. Individual nutritional treatments were calculated so that animals in the growth groups gained weight (100 g/day) and animals in the restricted groups lost weight (100 g/day). The trial lasted 42 days, after which animals were slaughtered in a commercial abattoir, following commercial practices. For further information, kindly refer to Scanlon *et al*.^10^ and Almeida *et al*.^21^.

By the end of the nutrition trial, gastrocnemius muscle and liver tissues were sampled and preserved at −80 °C for further analysis.

### Sample processing

For muscle tissue we analysed 11 samples in all experimental groups, with the exception of the Merino Restricted group where 10 samples were used. For the liver tissue we used 12 samples for all groups, with the exception of the Merino Restricted group where 11 samples were used. Frozen tissues were powdered individually with porcelain mortar and pestle with liquid nitrogen. Metabolites were extracted following the Bligh and Dyer method^22^ with adaptations. Muscle samples were processed as previously described for goat mammary gland samples^16^. For liver samples the solvent volumes used were modified as follows: an initial 3 ml cold chloroform/methanol mixture (1:2, v/v) was added to the tissue and mixed, followed by the addition and mixing of 2 ml of cold chloroform. Then 1 ml of cold water was added and mixed by vortexing. For both tissues, the mixture was finally centrifuged and the methanol/water fraction was separated and dried as previously described^16^.

### NMR Spectroscopy

The aqueous fraction of the muscle samples was re-suspended in 600 μl phosphate buffer (150 mM, pH 7.0/pD 7.4, with 1 mM sodium-2,2-dimethyl-2-silapentane-5-sulfone (DSS), in D_2_O), while the water-soluble fraction of the liver samples was dissolved in 800 μl phosphate buffer (100 mM, pH 7.4/pD 7.8, with 0.5 mM DSS, in D_2_O). Samples were transferred into 5 mm NMR tubes.

Proton (^1^H) NMR spectroscopy was conducted on an 800 MHz Bruker AvanceII^+^ (Ettlingen, Germany) spectrometer, with a triple resonance HCN Z-gradient probe, at 298 K.

^1^H 1D-NOESY spectra were collected for each sample using the “noesypr1d” pulse sequence (spectral width: 12 ppm; mixing time: 0.1 s; relaxation delay: 1 s; acquisition time: 4 s), following the parameters for profiling recommended from Chenomx NMR Suite software (Chenomx Inc., Edmonton, Canada). All spectra were processed with a line broadening (lb) of 0.5 Hz and a final number of 128 K points. Additional J-resolved spectra were collected to assist with assignment. All spectra were acquired, processed and analysed using TopSpin 3.2 (Bruker, Ettlingen, Germany).

### Metabolite Profiling

Metabolite identification and quantification was carried out using Chenomx NMR Suite 8.12 software (Chenomx Inc., Edmonton, Canada), using the internal reference library (Version 10), and with support of published data for other animals^23^,^24^,^25^.

### Data Analysis

Both univariate and multivariate analysis were performed for the obtained metabolite concentrations, following the approach previously described^16^. Briefly, for univariate analysis we performed a t-test with 2 tails and test type 3, considering *p* < 0.05 to reject null-hypothesis (equal means between groups), using Microsoft Excel. Multivariate analysis was performed using the SIMCA 13.0.3.0 software (Umetrics AB, Umeå, Sweden) for unsupervised Principal Components Analysis (PCA) and supervised Partial Least Squares Discriminant Analysis (PLS). In PLS analysis, Q^2^ (predictive ability of the model) and R^2^ (goodness of the fit) were considered as quality parameters of the model. Results were accepted for Q^2^ above 0.5^26^. For PLS models, a permutation test was additionally performed, using 100 permutations and accepting the model as “valid” when R^2^Y-intercept < 0.4 and Q^2^Y-intercept < 0.05. All ellipses in the scores plots were drawn at the 95% confidence level.

### Animal Welfare disclaimer

All work involving animals was conducted according to relevant international guidelines (European Union procedures on animal experimentation—Directive 2010/63/EU) that regulate the use of production animals in animal experimentation. These define that in the case of experiments carried out under standard production conditions, no approval from an ethics committee is required. Nevertheless, this experiment was conducted with the approval of the Ethics Committee of the Department of Agriculture and Food Western Australia (DAFWA, Perth, WA, Australia) registered as process 07ME06. The entire trial was conducted under the supervision of the veterinary authority in the State of Western Australia. Author AM Almeida holds a FELASA (Federation of European Laboratory Animal Society Associations) grade C certificate that enables designing and carrying out animal experimentation under European Union regulations. Animal management, handling, transport and slaughter were all conducted replicating approved standard commercial practices in the Commonwealth of Australia and in the State of Western Australia.

## Results

### Muscle tissue

**Figure 1:**
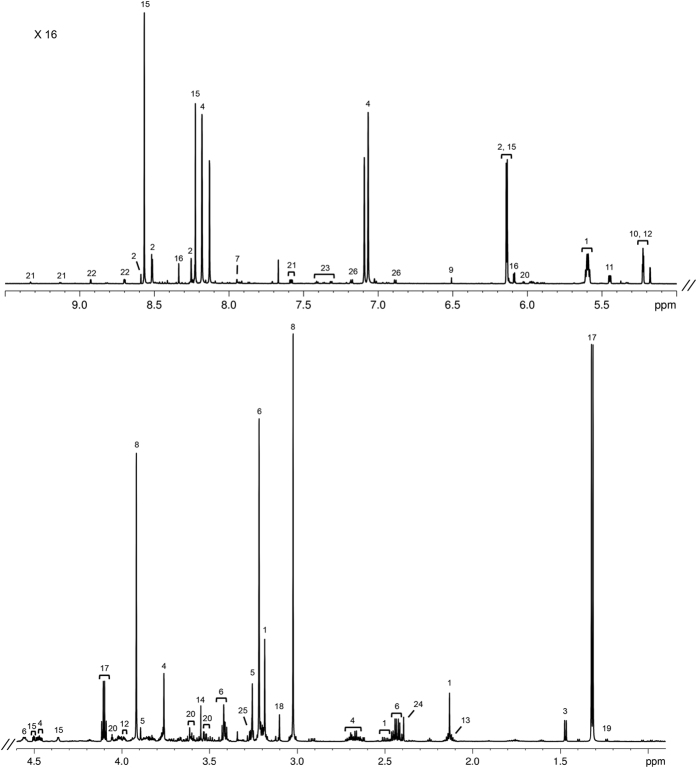
Representative 800 MHz 1H 1D NOESY spectrum of gastrocnemius muscle aqueous fraction in Merino breed, growth group. Key: (1) acetyl-L-carnitine; (2) ADP/AMP/ATP; (3) alanine; (4) anserine; (5) betaine; (6) carnitine; (7) carnosine; (8) creatine/creatine phosphate; (9) fumarate; (10) glucose; (11) glucose-1-phosphate; (12) glucose-6-phosphate; (13) glutamine; (14) glycine; (15) IMP; (16) inosine; (17) lactate; (18) malonate; (19) methylmalonate; (20) myo-inositol; (21) NAD^+^/NADP^+^; (22) nicotinurate; (23) phenylalanine; (24) succinate; (25) taurine; (26) tyrosine.

A representative ^1^H 1D NOESY spectrum of the sheep gastrocnemius muscle (Merino breed, growth group) is shown in Fig. 1.

A total of 51 metabolites were identified and quantified in the aqueous fractions of the muscle, and the average metabolite concentrations from the six experimental groups are shown in Supplementary Table S1. The most abundant metabolites are lactate and creatine/creatine-phosphate in all breeds, followed by taurine, anserine, carnitine and glutamine in the Dorper and Damara breeds; and carnitine, malonate, taurine and anserine in the Merino breed. Significant differences (*p* < 0.05) between growth and restricted groups for each breed are presented in Table 1. Among these, the more marked differences were observed in glycerophosphocholine in the Merino breed which decreased 4.1 times between growth and restricted groups; and adenine in the Dorper breed, which increased 2.5 times from growth to restricted groups. In the Merino breed, we identified differences between growth and restricted groups in citrate, glucose-6-phosphate, glutathione, glycerophosphocholine, glycine, acetyl-L-carnitine, taurine and tyrosine. In the Damara breed, differences between growth and restricted groups were observed in glucose-1-phosphate, inosine monophosphate (IMP), isoleucine, leucine, tyrosine, valine, phenylalanine and taurine. In the Dorper breed only four metabolites show significant differences between groups: adenine, formate, glycine and taurine.

**Figure 2:**
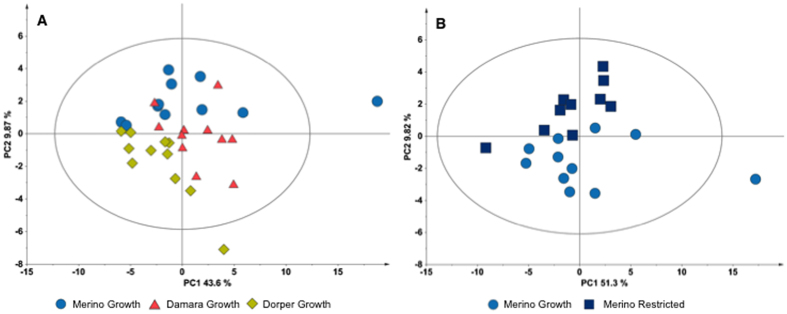
Multivariate analysis of gastrocnemius muscle (aqueous fraction) metabolites concentration. (**A**) PCA scores plot for the three growth groups (NC = 2, PC1 = 43.6%, PC2 = 9.87%); (**B**) PCA scores plot for Merino growth and restricted groups (NC = 2, PC1 = 51.3%, PC2 = 9.82%). Ellipse Hotelling’s T2 (95%).

Multivariate analysis was applied to metabolite concentrations. PCA scores plot of all groups does not show any specific separation by group (Supplementary Figure S1A), however it is possible to see some distinction by breed. In the PCA scores plot of the growth groups (Fig. 2A) it is possible to see some separation among breeds, especially between Merino and Dorper. On the other hand, the PCA scores plot of the restricted groups revealed no specific separation between breeds (Supplementary Figure S1B). However, it is noteworthy that the Damara restricted groups show a less broad distribution when compared to the distributions of the other two breeds. Multivariate analysis was also applied per breed. PCA scores of Merino growth and restricted groups revealed the two groups could be separated by the second principal component (PC2) (Fig. 2B). Analysis of the loadings (Supplementary Table S2) indicate that the metabolites that contribute more to this separation were glycerophosphocholine, citrate, acetyl-L-carnitine, myo-inositol, glutathione and glucose-6-phosphate. For Damara and Dorper breeds it was only possible to separate their growth and restricted groups applying PLS analysis. Damara growth and restricted groups were separated by the first principal component (PC1) with acceptable quality parameters (Supplementary Figure S1C). However, the permutation test (Supplementary Figure S1D) failed the model validation. Concerning the Dorper growth and restricted groups, PLS was not able to separate the groups with acceptable quality parameters (Supplementary Figure S1E). Furthermore, permutation test (Supplementary Figure 1F) do not validate the model.

### Liver tissue

**Figure 3:**
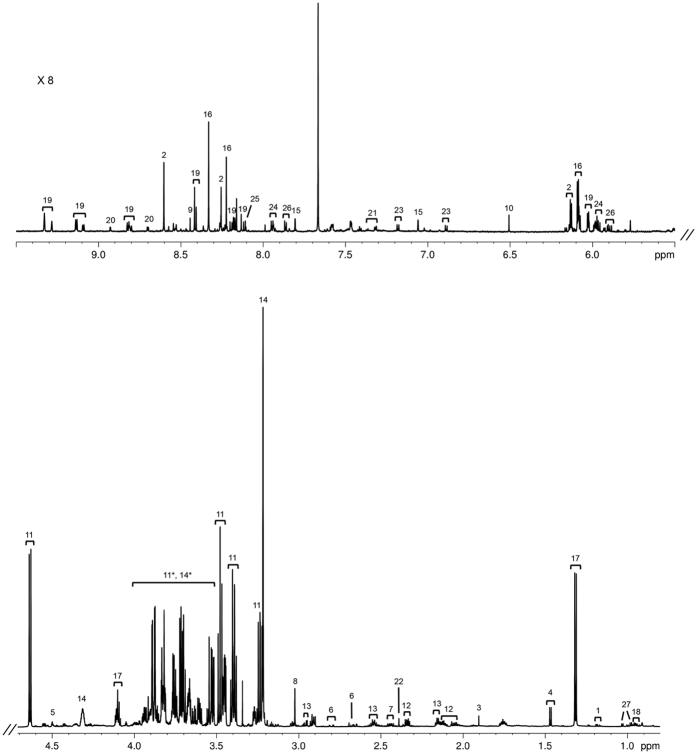
Representative 800 MHz 1H 1D NOESY spectrum of liver aqueous fraction in Merino breed, growth group. Key: (1) 3-hydroxybutyrate; (2) ADP/AMP/ATP; (3) acetate; (4) alanine; (5) ascorbate; (6) aspartate; (7) carnitine; (8) creatine/creatine phosphate; (9) formate; (10) fumarate; (11) glucose; (12) glutamate; (13) glutathione; (14) glycerophosphocholine; (15) histamine; (16) inosine; (17) lactate; (18) leucine; (19) NAD^+^/NADP^+^/NADPH; (20) nicotinurate; (21) phenylalanine; (22) succinate; (23) tyrosine; (24) UDP-glucose/UDP-glucoronate; (25) UMP; (26) uridine; (27) valine; (11*, 14*) region strongly dominated by glucose and glycerophosphocholine peaks, but with some less concentrated metabolites underneath them.

A representative ^1^H 1D NOESY spectrum of the sheep liver (Merino breed, growth group) was selected and is shown in Fig. 3, with examples of some of the identified metabolites.

A total of 46 metabolites were identified in the aqueous fraction of sheep liver and the metabolite concentration of all experimental groups are presented in Supplementary Table S3. The most abundant metabolites are glucose, lactate, glycerophosphocholine and glutamate in the three breeds, followed by taurine, glycine, glutathione and alanine in different orders depending on the breed. Univariate analysis revealed differences between growth and restricted groups (Table 2) of the Merino breed in 3-hydroxybutyrate, acetate, alanine, ascorbate, creatine/creatine-phosphate, lactate, sarcosine, succinate, glutathione, and UDP-glucose/UDP-glucoronate. In the Damara breed, differences between growth and restricted groups are identified in acetate, adenine, alanine, ascorbate, choline/acetylcholine/phosphocholine, citrate, fomate, lactate and UDP-glucose/UDP-glucoronate. It is noteworthy to mention that in the Dorper breed only two metabolites: carnitine and sarcosine, show differences between growth and restricted groups.

**Figure 4:**
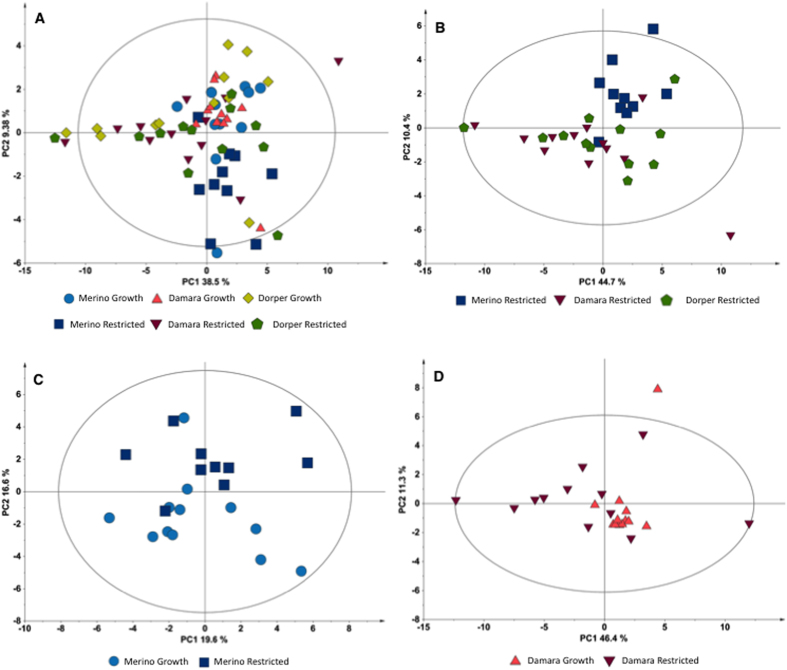
Multivariate analysis of liver (aqueous fraction) metabolites concentration. (**A**) PCA scores plot for the six experimental groups (NC = 4, PC1 = 38.5%, PC2 = 9.38%); (**B**) PCA scores plot for the three restricted groups (NC = 5, PC1 = 44.7%, PC2 = 10.4%); (**C**) PCA scores plot for Merino growth and restricted groups (NC = 2, PC1 = 19.6%, PC2 = 16.6%); (**D**) PCA scores plot of Damara growth and restricted groups (NC = 3, PC1 = 46.4%, PC2 = 11.3%). The ellipses in the scores plots are drawn at the 95% confidence level. Ellipse Hotelling’s T2 (95%).

Concerning the multivariate analysis, albeit the PCA scores plot of the six experimental groups (Fig. 4A) does not reveal a clear separation of the groups, some tendencies may be noted. Dorper growth and restricted group have indistinguishable distribution, whereas the Merino groups seem to be separated into clusters influenced by the PC2. This separation of the Merino groups from the other groups could be due to variations in benzoate, glycine and succinate, as shown in loadings list (Supplementary Table S4). PCA scores plot of the three restricted groups (Fig. 4B) show a distinct clustering of the Merino restricted samples away from the other two restricted groups of Damara and Dorper breeds. Loadings list of this model indicates that separation is due to variations in succinate, formate, and xanthine (Supplementary Table S5). Merino growth and restricted groups show separation in the PCA scores plot by the PC2 (Fig. 4C). Loading list of this model (Supplementary Table S6) indicate variations in glycine, formate, lactate and succinate. PLS analysis for this breed (Merino growth and restricted groups) has good quality parameters but the permutation test does not validate the model. PCA scores plot of the Damara growth and restricted groups (Fig. 4D) show a slight separation in the PC1. The growth group have less disperse distributions whereas the restricted group is more spread-out along the plot area. Separation of the two groups could be due to differences in glucose, tyrosine, isoleucine, inosine and leucine (Supplementary Table S7). PLS analysis for the two groups of Damara breed has unacceptable quality parameters and cannot be validate by the permutation test. The Dorper growth and restricted groups (Supplementary Figure S2A) were only separated by the PLS analysis. Although the quality parameters are acceptable, the permutation test does not validate the model (Supplementary Figure S2B).

## Discussion

To the best of our knowledge, this work is the first to apply the NMR technique to studies on the effects of SWL in muscle and liver metabolome on the Merino, Damara and Dorper sheep breeds. The approach proved to be adequate for the study, allowing the identification and quantification of 51 metabolites in muscle, and 46 in liver.

As suggested for other metabolomics studies^27^, we analysed the data using both univariate and multivariate analyses. PCA of the different groups did not reveal a clear separation between groups, neither for muscle nor for liver, although the muscle samples could be discriminated by breed. In the per-breed analysis, some tendencies are noted, especially in the Dorper breed, where it was impossible to discriminate between the two nutritional treatment groups with good quality parameters, in both liver and muscle tissues. Such separation was possible for the Merino and Damara in both tissue samples. These results could be an indicator of a more pronounced reaction of the muscle to feed-restriction in Merino and Damara, and a general different response in the Dorper breed.

Univariate analysis results of the Dorper growth and restricted groups revealed that few metabolite variations were found in both tissues, four in muscle and two in liver. In the Merino and Damara breeds, the extent of metabolite differences in both tissues was comparable, with half of them coinciding in both breeds. The Merino had eight metabolites with variations in muscle and 10 in liver tissue, while the Damara had eight in muscle and nine in liver.

**Figure 5:**
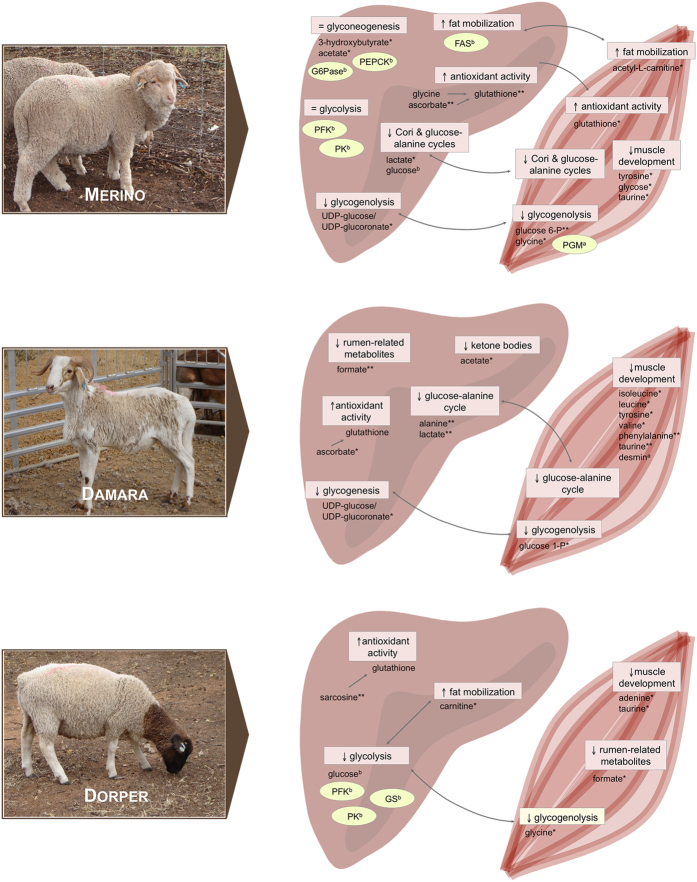
Schematic representation of major results in muscle and liver metabolome of restricted groups, of Merino, Damara and Dorper breeds, when compared with the respective growth group. Results from previous work in the same animals are also shown, and marked with uppercase letters (a/b). Metabolites with significant variation are marker with stars (*/**). Key: (**a**) Almeida *et al*. 2016. PLoS One. 11(2):e0146367 (Reference no. 13); (**b**) van Harten *et al*. 2013. Animal. 7:439–445 (Reference no. 12); (*) p < 0.05; (**) p < 0.01; (FAS) fatty acid synthase; (G6Pase) glucose 6-phosphatase; (PEPCK) phosphoenolpyruvate carboxykinase; (PFK) phosphofructokinase; (PK) pyruvate kinase; (PGM) phosphoglucomutase; (GS) glycogen synthase; (=) no variation in the pathway; (↑) pathway increased; (↓) pathway decreased.

The distinct variations among breeds, identified by both statistical approaches, could be indicative of breed-specific response to the feed-restriction treatment. Interestingly, we observed that some muscle metabolites could explain differences between treatment groups in both statistical analyses in Merino and Damara. In the liver, the same was observed for the Merino breed. However, a more detailed analysis of these variations is needed to understand the physiological significance of these observations. Figure 5 summarises the major results for each breed and includes some results from previous studies on these animals, to help integrate and evaluate all the information.

### Merino breed metabolomes

In muscle samples, decreased levels of amino acids like tyrosine, glycine and taurine during restriction could be an indication of a reduction of muscle growth. Taurine levels can also be affected by a reduction in dietary cysteine levels^28^, making it difficult to separate the effect of diet restriction and the inner response of the animal.

Glycine also has an additional role as a precursor of glucagon in glycogenolysis^29^, and can affect glutathione production^30^,^31^ in the antioxidant defence mechanism. However, glutathione synthesis can also be limited by diet related cysteine intake^32^, mixing the effects of the restricted feed with those of the metabolic response. Since glutathione is produced in liver and released to the muscle^33^, its decreased levels in the liver could be related with its increase in muscle of the same animals. The lower levels of ascorbate (Vitamin C) in liver could also be related to glutathione levels^34^. However, ruminants are able to synthesize it from glucose, and its reduction could also be related to the diet restriction. Low levels of glutathione and ascorbate could be indicative of oxidative stress in the tissue^35^ likely as a consequence of weight loss. Sarcosine is a precursor of creatine, so variations in their concentrations in liver could be related with this pathway^36^,^37^. Levels of creatine/creatine-phosphate, two metabolites related to energy production in muscle, are in fact increased in the restricted merino group as well as in the other breeds. As previously mentioned, glycine is a precursor of glucagon, which, during low glucose levels and under stress conditions, promotes gluconeogenesis and glycogenolysis. Indeed, significant differences in some metabolites related with such these metabolic pathways were observed. Glucose-6-phosphate and UDP-glucose/UDP-glucoronate are intermediate products in the glycogenolysis pathway. Their lower levels in muscle of the restricted group could be due to the depletion of glucose and glycogen stock in these animals, due to the diet limitation. Previous proteome analysis of the same muscle samples revealed an increased expression of phosphoglucomutase, an enzyme involved in glycogenolysis and glycogenesis^13^. The over expression occurs only in the Merino breed and not in the SWL tolerant breeds, confirming that this breed is more susceptible to feed restriction and has a specific response to this treatment. van Harten *et al*.^12^ determined the gene expression of regulatory enzymes in the liver of the same animals (Dorper and Merino), and no changes were observed in the enzymes of the gluconeogenesis (phosphoenolpyruvate carboxylase) and glycolysis (phosphofructokinase and pyruvate kinase) pathways. The expression level of glucose-6-phosphatase, essential in glucose supply during feed-restriction, was determined and its value decreased in restricted groups. During diet restriction animals tend to reduce the glycolytic pathway and promote gluconeogenesis^12^. These results could then be indicative of a minor adaptation to the feed-restriction.

In the livers of the restricted group we also observed lower levels of alanine and lactate. These two metabolites are related in both muscle and liver to the Cori and the Alanine-Glucose Cycles. A decrease on their concentrations could be an indirect consequence of the lower levels of glucose in both tissues. Alanine is also a structural amino acid and its concentration is usually low during diet restriction when muscle breakdown occurs. Glucose levels in the liver of the same animals were determined in a previous study and was observed a significant decrease between growth and restricted animals^12^, supporting this hypothesis.

Energy from nutrients in the diet could be obtained through processes other than glycolysis, especially during feed-restriction periods. Variations in some metabolites are indicative of such process. The increased levels of acetyl-L-carnitine in the muscle of the restricted merino group is an indication of fat mobilization. This metabolite is responsible for the transport of fatty acids into mitochondria to be oxidized and used as energy sources during high energy demanding or glucose starvation periods. Previous results showed lower expression levels of fatty acid synthase, an enzyme responsible for fatty acids synthesis, in the liver of restricted animals^12^. These results suggest that restricted-fed animals are using fatty acids as an energy source.

During fasting periods and low carbohydrate diets, ketone bodies are produced in liver through gluconeogenesis. We present some indicators of ketone body production in the liver of the restricted group, as 3-hydroxybutyrate and acetate, derived respectively from acetone and acetoacetate. However, in ruminant both compounds could be a result of rumen activity^16^,^38^ due either to changes in microflora profile or in the diet composition.

Succinate and citrate show variations between groups in liver and muscle, respectively. Both metabolites are intermediates in the Krebs cycle with associations to other metabolic pathways. These variations could be indicative of variations in the Krebs cycle or regulation of other secondary pathway, as the inhibition of glycolysis and promotion of gluconeogenesis by elevated levels of citrate. The increased levels of glycerophosphocholine in the muscle could also be related with some regulatory process, since this metabolite is a storage form of choline in cytosol, with functions as muscle control and source of methyl group^39^.

It is noteworthy that, as wool producers these animal will use nutritional resources mainly or wool production, making them unavailable to be used in other pathways in harsh conditions. Also, the wool production continued during the trial, channelling nutritional resources to it and influencing the general pathways.

### Damara breed metabolomes

Levels of isoleucine, leucine, tyrosine, valine, phenylalanine and taurine decreased in muscle of the restricted group. All these amino acids are related to muscle development and their lower concentrations could be directly linked to muscle production decrease. However, a previous study on muscle of these animals revealed a unique individual response of this breed, when compared with the Merino and Dorper breeds^13^. In the Damara breed the levels of desmin, a muscle-specific protein responsible for cell architecture, increased, ensuring the structure and function of the muscle even if some tissue mobilization occurs^13^. Isoleucine and leucine are also related with other metabolic pathways, related to ketonic bodies production and cell growth regulator respectively, that can influence their concentrations. The reduction of UDP-glucose/UDP-gucoronate and glucose-1-phosphate in liver and muscle respectively, as observed in the Merino breed, is indicative of changes in the glycogenolysis/glycogenesis pathway.

IMP and adenine levels are both lower in restricted group, in muscle and liver respectively. IMP is a nucleoside and an intermediate in purine metabolism, from which adenine is one of the examples. This result could be a consequence of a slower muscle development or an imbalance in these tissues. Choline could also be connected with cell development and balance^39^, and it is also reduced in the liver of the restricted-fed animals. Variations in Krebs cycle intermediates (citrate), in rumen-related metabolite (formate), and in ascorbate was observed in this breed, similarly to the Merino breed.

Considering the special adaptation of this breed to store fat in the tail, it is interesting that changes observed in the metabolism are in general not directly related to fat metabolism. Previous studies with the same animal groups suggest that the Damara breed has a unique lipid metabolism, mostly due to the putative contribution of the fat tail as supplier of odd and branched-chain fatty acids (BCFA) to the muscle^40^. However, it is also suggested that this tolerance to feed restriction could also be due to some kind of peculiarities in rumen activity^40^. Specifically, if the Damara breed has some digestive adaptation that can increase the efficiency of fibre digestion, the acetate-propionate ratio will be affected^40^,^41^. Indeed, in the present study, levels of acetate in the liver of the restricted group was lower than in the growth group.

### Dorper breed metabolomes

Variations in glycine and sarcosine in this breed show the same pattern as observed in Merino breed. Higher levels of sarcosine in liver of the restricted groups could be indicative of an increase glutathione production. At the same time, lower levels of glycine in muscle of restricted groups suggest a decrease of glycogenolysis. Previous results^12^ on the enzyme expression levels show that enzymes related with glycolysis (phosphofructokinase and pyruvate kinase) have lower expression in the restricted group. Simultaneously, enzyme related to glycogenesis (glycogen synthase) did not vary between treatments. Moreover, the same study showed that levels of glucose in liver of restricted group did not differ from the values of growth group^12^, suggesting an efficient response of this breed to feed restriction condition.

Since carnitine is essential for fat mobilization and energy production during fasting periods and feed restriction, higher levels of this metabolite in the liver could help explain the glucose homeostasis of this breed. Previous results on the enzymes related with fatty acid synthesis (fatty acid synthase)^12^ revealed a decrease on its expression in the restricted group, reinforcing the hypothesis of fatty acids request for energy production.

As observed in the other two breeds, taurine levels are lower in the muscle of the restricted group, being indicative of lower muscle production and development. However, previous results on enzymes involved in protein catabolism (glutamate dehydrogenase) show a decrease in restricted groups^12^. These results suggest that in the Damara muscle production could be reduced due to feed restriction, while the Dorper breed can maintain tissue function and structure. Adenine concentration was higher in muscle of the restricted group, opposite to what was observed in the liver of the Damara breed. This metabolite is a nucleoside with functions in protein synthesis and energy production.

The Dorper breed also showed differences in rumen-related metabolites (formate) between the restricted and growth groups that could be indicative of adaptations in rumen microbiota as response to the restriction-fed regime.

### Metabolomics results, growth, carcass traits, leptin and insulin concentrations

In general, we observed a decrease in muscle development, an increase in antioxidant activity and differences in energy production pathways between the different breeds as each breed cope with feed restriction. Dorper and Damara breeds seem to be more tolerant to feed restriction. Previous studies on these animals^7^,^13^ (Supplementary Table S8) already suggested this tendency. Variations in the carcass weight and yields and in the dimensions of eye muscle were similar in Dorper and Damara breed, and both different of what was observed in Merino. Concerning plasma parameters, Damara breed presented differences in leptin and insulin concentrations, when compared with the other breeds. Higher levels of leptin and insulin in Damara could be justified by the existence of the tail fat depot. Leptin is produced by adipose cells and is highly correlated with body fat, whereas insulin stimulates lipogenesis and fatty acids esterification^42^. In ruminants, insulin stimulates the leptin expression^43^, which together with the body fat content, could explain the higher level of leptin concentrations in this breed.

### Conclusions and future prospects

In general, the Dorper breed seems to be more adapted to SWL, showing few changes in both tissues when subjected to feed restriction. This tolerance could be a result of the breed-selection history. The Merino, probably due to its selection for wool production, showed more marked changes both tissues and seems to be the less adapted to SWL. The Damara presented a specific set of adaptations, reflecting the physiology of its major body characteristic, the fat-tail. In the context of the breed selection towards SWL tolerance, our results confirm that the Dorper and Damara breeds have performed better under SWL conditions. Their adaptation seems to be linked to a more efficient metabolic adaptation to feed-restriction, which change the nutritional energy source without compromising the overall muscle structure. A possible adaptation at the rumen level should also be considered in these breeds, since they presented some variations related with rumen microflora composition and activity.

## Additional Information

**How to cite this article**: Palma, M. *et al*. The hepatic and skeletal muscle ovine metabolomes as affected by weight loss: a study in three sheep breeds using NMR-metabolomics. *Sci. Rep.* **6**, 39120; doi: 10.1038/srep39120 (2016).

**Publisher's note:** Springer Nature remains neutral with regard to jurisdictional claims in published maps and institutional affiliations.

## Supplementary Material

Supplementary Material

## Figures and Tables

**Table 1:** Metabolites in gastrocnemius muscle of Merino, Dorper and Damara sheep breeds, with significant differences between growth and restricted groups, in at least one breed.

	Merino		Dorper		Damara	
	growth	restricted	growth	restricted	growth	restricted
Acetyl-L-carnitine	2.6 E-03 ± 1.2 E-03	4.6 E-03* ± 1.9 E-03	1.6 E-03 ± 5.8 E-04	1.6 E-03 ± 1.0 E-03	2.2 E-03 ± 3.8 E-04	2.2 E-03 ± 7.6 E-04
Adenine	8.3 E-05 ± 7.5 E-05	7.2 E-05 ± 5.0 E-05	2.1 E-05 ± 1.1 E-05	5.3 E-05* ± 4.1 E-05	8.5 E-05 ± 4.3 E-05	7.4 E-05 ± 6.5 E-05
Citrate	1.7 E-04 ± 8.6 E-05	2.7 E-04* ± 1.1 E-04	1.5 E-04 ± 7.5 E-05	1.5 E-04 ± 1.2 E-04	1.9 E-04 ± 7.9 E-05	2.2 E-04 ± 6.8 E-05
Formate	5.3 E-05 ± 2.0 E-05	4.0 E-05 ± 7.1 E-06	4.8 E-05 ± 1.7 E-05	3.5 E-05* ± 8.2 E-06	4.7 E-05 ± 1.4 E-05	5.6 E-05 ± 1.1 E-05
Glucose-1-phosphate	6.3 E-04 ± 2.7 E-04	5.2 E-04 ± 2.0 E-04	5.0 E-04 ± 1.5 E-04	4.6 E-04 ± 3.2 E-04	7.0 E-04 ± 2.1 E-04	4.8 E-04* ± 2.7 E-04
Glucose-6-phosphate	2.9 E-03 ± 1.2 E-03	1.6 E-03** ± 7.3 E-04	2.7 E-03 ± 6.3 E-04	1.9 E-03 ± 1.2 E-03	3.1 E-03 ± 1.1 E-03	2.2 E-03 ± 9.7 E-04
Glutathione	1.7 E-04 ± 1.0 E-04	2.8 E-04* ± 1.3 E-04	2.2 E-04 ± 6.6 E-05	2.5 E-04 ± 1.3 E-04	2.3 E-04 ± 9.6 E-05	3.1 E-04 ± 1.6 E-04
Glycerophosphocholine	3.1 E-04 ± 1.1 E-04	7.7 E-05** ± 2.4 E-05	3.8 E-04 ± 1.9 E-04	5.7 E-04± 3.0 E-04	4.0 E-04 ± 1.7 E-04	5.5 E-04 ± 1.8 E-04
Glycine	3.5 E-03 ± 1.7 E-03	1.9 E-03* ± 5.4 E-04	2.8 E-03 ± 7.7 E-04	1.9 E-03* ± 1.3 E-03	2.2 E-03 ± 4.4 E-04	1.8 E-03 ± 7.0 E-04
IMP	2.5 E-03 ± 1.1 E-03	1.9 E-03 ± 7.4 E-04	1.5 E-03 ± 6.5 E-04	1.7 E-03 ± 8.6 E-04	2.5 E-03 ± 7.0 E-04	1.7 E-03* ± 7.7 E-04
Isoleucine	9.3 E-05 ± 3.9 E-05	7.8 E-05 ± 2.2 E-05	8.6 E-05 ± 2.4 E-05	8.2 E-05 ± 4.8 E-05	1.1 E-04 ± 1.4 E-05	9.1 E-05* ± 2.5 E-05
Leucine	1.4 E-04 ± 5.6 E-05	1.1 E-04 ± 2.6 E-05	1.3 E-04 ± 3.7 E-05	1.2 E-04 ± 5.3 E-05	1.6 E-04 ± 2.2 E-05	1.3 E-04* ± 3.1 E-05
Phenylalanine	4.9 E-05 ± 1.6 E-05	3.9 E-05 ± 1.1 E-05	4.4 E-05 ± 9.5 E-06	3.5 E-05 ± 1.3 E-05	5.3 E-05 ± 8.2 E-06	4.0 E-05** ± 8.6 E-06
Taurine	5.4 E-03± 3.3 E-03	3.0 E-03* ± 1.1 E-03	6.3 E-03 ± 2.8 E-03	3.9 E-03* ± 2.4 E-03	1.4 E-02 ± 4.4 E-03	8.8 E-03** ± 2.0 E-03
Tyrosine	5.5 E-05 ± 2.7 E-05	3.5 E-05* ± 1.3 E-05	5.0 E-05 ± 1.7 E-05	3.8 E-05 ± 1.6 E-05	6.3 E-05 ± 1.6 E-05	4.6 E-05* ± 1.2 E-05
Valine	2.1 E-04 ± 10.0 E-05	1.5 E-04 ± 3.5 E-05	2.0 E-04 ± 5.7 E-05	1.9 E-04 ± 1.1 E-04	2.3 E-04 ± 4.0 E-05	1.9 E-04* ± 3.7 E-05

Average concentration (mmol/g tissue) and standard deviation are shown for each experimental group.

Key: *p < 0.05 when compared with growth groups of the same breed; **p < 0.01 when compared with growth groups of the same breed.

**Table 2:** Metabolites in liver of Merino, Dorper and Damara sheep breeds, with significant differences between growth and restricted groups, in at least one breed.

	Merino		Dorper		Damara	
	growth	restricted	growth	restricted	growth	restricted
3-Hydroxybutyrate	3.3 E-04 ± 1.0 E-04	4.7 E-04* ± 1.4 E-04	2.4 E-04 ± 10.0 E-05	3.2 E-04 ± 1.4 E-04	2.9 E-04 ± 7.9 E-05	2.7 E-04 ± 1.4 E-04
Acetate	5.9 E-04 ± 1.3 E-04	8.3 E-04** ± 1.7 E-04	4.9 E-04 ± 2.3 E-04	5.8 E-04 ± 2.5 E-04	6.1 E-04 ± 1.3 E-04	4.3 E-04* ± 2.3 E-04
Adenine	3.3 E-04 ± 1.5 E-04	3.0 E-04 ± 1.6 E-04	1.6 E-04 ± 9.7 E-05	1.9 E-04 ± 1.0 E-04	3.0 E-04 ± 4.5 E-05	1.6 E-04** ± 1.2 E-04
Alanine	2.0 E-03 ± 4.9 E-04	1.4 E-03** ± 4.2 E-04	1.8 E-03 ± 8.8 E-04	1.4 E-03 ± 4.6 E-04	2.4 E-03 ± 2.8 E-04	1.5 E-03** ± 7.2 E-04
Ascorbate	1.2 E-03 ± 3.7 E-04	7.7 E-04** ± 3.5 E-04	9.2 E-04 ± 5.0 E-04	7.2 E-04 ± 3.2 E-04	1.2 E-03 ± 2.5 E-04	8.2 E-04* ± 4.1 E-04
Carnitine	5.7 E-04 ± 3.2 E-04	5.8 E-04 ± 3.5 E-04	6.4 E-04 ± 3.5 E-04	9.7 E-04* ± 4.3 E-04	7.0 E-04 ± 2.6 E-04	7.1 E-04 ± 3.8 E-04
Choline/Acetylcholine/Phosphocholine	6.0 E-04 ± 3.0 E-04	8.8 E-04 ± 6.6 E-04	4.6 E-04 ± 3.6 E-04	5.4 E-04 ± 2.7 E-04	6.2 E-04 ± 2.6 E-04	3.4 E-04* ± 2.3 E-04
Citrate	3.6 E-04 ± 1.2 E-04	2.6 E-04 ± 1.3 E-04	3.2 E-04 ± 1.4 E-04	2.5 E-04 ± 1.1 E-04	4.9 E-04 ± 1.2 E-04	2.9 E-04** ± 1.8 E-04
Creatine/Creatine phosphate	1.7 E-03 ± 5.2 E-04	2.6 E-03** ± 7.2 E-04	1.5 E-03 ± 6.8 E-04	1.8 E-03 ± 8.3 E-04	1.6 E-03 ± 3.3 E-04	1.7 E-03 ± 1.0 E-03
Formate	1.2 E-04 ± 7.6 E-05	1.3 E-04 ± 5.6 E-05	3.9 E-05 ± 3.4 E-05	5.0 E-05 ± 5.0 E-05	1.2 E-04 ± 5.6 E-05	4.8 E-05** ± 2.5 E-05
Glutathione	2.5 E-03 ± 7.4 E-04	1.2 E-03** ± 4.3 E-04	2.2 E-03 ± 1.4 E-03	1.5 E-03 ± 6.4 E-04	2.5 E-03 ± 4.8 E-04	1.9 E-03 ± 1.3 E-03
Lactate	2.3 E-02 ± 4.4 E-03	2.0 E-02*± 3.0 E-03	1.7 E-02 ± 7.8 E-03	1.5 E-02± 5.3 E-03	2.1 E-02 ± 2.7 E-03	1.5 E-02** ± 5.3 E-03
Sarcosine	2.5 E-05 ± 1.1 E-05	6.6 E-05* ± 4.7 E-05	1.9 E-05 ± 9.9 E-06	5.0 E-05** ± 3.3 E-05	2.5 E-05 ± 9.9 E-06	2.8 E-05± 1.9 E-05
Succinate	1.9 E-03 ± 7.0 E-04	1.1 E-03* ± 7.4 E-04	1.4 E-03 ± 9.4 E-04	1.0 E-03 ± 5.2 E-04	1.6 E-03 ± 6.4 E-04	1.4 E-03 ± 6.2 E-04
UDP-glucose/UDP-glucoronate	2.4 E-04 ± 6.0 E-05	1.7 E-04* ± 6.7 E-05	1.8 E-04 ± 8.4 E-05	2.0 E-04 ± 8.6 E-05	1.8 E-04 ± 4.4 E-05	1.4 E-04* ± 5.2 E-05

Average concentration (mmol/g tissue) and standard deviation are shown for each experimental group.

Key: *p < 0.05 when compared with growth groups of the same breed; **p < 0.01 when compared with growth groups of the same breed.

## References

[b1] Boutonnet J. P. Perspectives of the sheep meat world market on future production systems and trends. Small Rum Res 189–195, doi: 10.1016/S0921-4488(99)00072-3 (1999).

[b2] Almeida A. The Damara in the context of Southern Africa fat-tailed sheep breeds. Trop Anim Health Pro 43, 1427–1441 (2011). 10.1007/s11250-011-9868-3 21509451

[b3] Almeida A. M., Schwalbach L. M. J., Cardoso L. A. & Greyling J. P. C. Scrotal, testicular and semen characteristics of young Boer bucks fed winter veld hay: The effect of nutritional supplementation. Small Rum Res 216–220, doi: 10.1016/j.smallrumres.2007.02.001 (2007).

[b4] Almeida A., Schwalbach L., Waal H., Greyling J. & Cardoso L. The effect of supplementation on productive performance of Boer goat bucks fed winter veld hay. Trop Anim Health Prod 38, 443–449 (2006). 17165615 10.1007/s11250-006-4368-6

[b5] De Almeida A. & Cardoso L. Animal production and genetic resources in Guinea Bissau: I – Northern Cacheu Province. Trop Anim Health Prod 529–536, doi: 10.1007/s11250-008-9130-9 (2008). 18716910

[b6] Almeida A. & Cardoso L. Animal production and genetic resources in Guinea Bissau: II–Tombali province. Trop Anim Health Pro 40, 537–543 (2008). 10.1007/s11250-008-9131-8 18716911

[b7] Almeida A. M. . Assessing carcass and meat characteristics of Damara, Dorper and Australian Merino lambs under restricted feeding. Trop Anim Health Prod 45, 1305–11 (2013). 23345065 10.1007/s11250-013-0361-z

[b8] Lérias J. R. . Body live weight and milk production parameters in the Majorera and Palmera goat breeds from the Canary Islands: influence of weight loss. Trop Anim Health Prod 45, 1731–6 (2013). 23712398 10.1007/s11250-013-0423-2

[b9] Lérias J. R. . Establishment of the biochemical and endocrine blood profiles in the Majorera and Palmera dairy goat breeds: the effect of feed restriction. J. Dairy Res. 82, 416–25 (2015). 26290160 10.1017/S0022029915000412

[b10] Scanlon T. . Live weight parameters and feed intake in Dorper, Damara and Australian Merino lambs exposed to restricted feeding. Small Ruminant Res 109, 101–106 (2013).

[b11] Tshabalala P. A., Strydom P. E., Webb E. C. & de Kock H. L. Meat quality of designated South African indigenous goat and sheep breeds. Meat Sci. 65, 563–70 (2003). 22063250 10.1016/S0309-1740(02)00249-8

[b12] Van Harten S. . Gene expression of regulatory enzymes involved in the intermediate metabolism of sheep subjected to feed restriction. Animal 7, 439–45 (2013). 23031388 10.1017/S1751731112001589

[b13] Almeida A. M. . The Effect of Weight Loss on the Muscle Proteome in the Damara, Dorper and Australian Merino Ovine Breeds. PLoS ONE 11, e0146367 (2016). 26828937 10.1371/journal.pone.0146367 PMC4734549

[b14] Van Harten S. . Fatty acid composition of the ovine longissimus dorsi muscle: effect of feed restriction in three breeds of different origin. J. Sci. Food Agric. 96, 1777–82 (2016). 26037039 10.1002/jsfa.7285

[b15] Webb E. C., Casey N. H. & Van Niekerk W. A. Fatty acids in the subcutaneous adipose tissue of intensively fed SA Mutton Merino and Dorper wethers. Meat Sci. 38, 123–31 (1994). 22059614 10.1016/0309-1740(94)90101-5

[b16] Palma M. . NMR-metabolomics profiling of mammary gland secretory tissue and milk serum in two goat breeds with different levels of tolerance to seasonal weight loss. Mol Biosyst 12, 2094–107 (2016). 27001028 10.1039/c5mb00851d

[b17] Xu C.. (1)H-Nuclear Magnetic Resonance-Based Plasma Metabolic Profiling of Dairy Cows with Fatty Liver. Asian-australas. J. Anim. Sci. 29, 219–29 (2016). 26732447 10.5713/ajas.15.0439 PMC4698702

[b18] Scano P. . (1)H NMR brain metabonomics of scrapie exposed sheep. Mol Biosyst 11, 2008–16 (2015). 25959287 10.1039/c5mb00138b

[b19] Mickiewicz B. . Metabolic profiling of synovial fluid in a unilateral ovine model of anterior cruciate ligament reconstruction of the knee suggests biomarkers for early osteoarthritis. J. Orthop. Res. 33, 71–7 (2015). 25283885 10.1002/jor.22743

[b20] Wang L. F. . The effects of acute lipopolysaccharide challenge on dairy goat liver metabolism assessed with (1) HNMR metabonomics. J Anim Physiol Anim Nutr (Berl). doi: 10.1111/jpn.12439 (2016). 26847913

[b21] Almeida A. M. . Assessing carcass and meat characteristics of Damara, Dorper and Australian Merino lambs under restricted feeding. Trop Anim Health Prod 45, 1305–1311 (2013). 23345065 10.1007/s11250-013-0361-z

[b22] Bligh E. G. & Dyer W. J. A Rapid Method of Total Lipid Extraction and Purification. Can J Biochem Physiol 37, 911–917 (1959). 13671378 10.1139/o59-099

[b23] Nicholas P., Kim D., Crews F. & Macdonald J. 1H NMR-based metabolomic analysis of liver, serum, and brain following ethanol administration in rats. Chem Res Toxicol 21, 408–20 (2007). 18095657 10.1021/tx700324t

[b24] Serkova N. . Metabolic profiling of livers and blood from obese Zucker rats. J Hepatol 44, 956–62 (2005). 16223541 10.1016/j.jhep.2005.07.009

[b25] Martínez-Granados B. . Metabolic profile of chronic liver disease by NMR spectroscopy of human biopsies. Int J Mol Med 27, 111–7 (2010). 21072494 10.3892/ijmm.2010.563

[b26] Triba M. N. . PLS/OPLS models in metabolomics: the impact of permutation of dataset rows on the K-fold cross-validation quality parameters. Mol Biosyst 11, 13–9 (2015). 25382277 10.1039/c4mb00414k

[b27] Saccenti E., Hoefsloot H., Smilde A., Westerhuis J. & Hendriks M. Reflections on univariate and multivariate analysis of metabolomics data. Metabolomics, doi: 10.1007/s11306-013-0598-6 (2014).

[b28] Stipanuk M. H. Role of the liver in regulation of body cysteine and taurine levels: a brief review. Neurochem. Res. 29, 105–10 (2004). 14992268 10.1023/b:nere.0000010438.40376.c9

[b29] Li C. . Regulation of glucagon secretion in normal and diabetic human islets by γ-hydroxybutyrate and glycine. J Biol Chem 3938–51, doi: 10.1074/jbc.m112.385682 (2012). 23266825 PMC3567647

[b30] Jackson A. A. The glycine story. Eur J Clin Nutr 45, 59–65 (1991). 2050089

[b31] Ruiz-Ramírez A., Ortiz-Balderas E., Cardozo-Saldaña G., Diaz-Diaz E. & El-Hafidi M. Glycine restores glutathione and protects against oxidative stress in vascular tissue from sucrose-fed rats. Clin. Sci. 126, 19–29 (2014). 23742196 10.1042/CS20130164

[b32] Stipanuk M. H., Coloso R. M., Garcia R. A. & Banks M. F. Cysteine concentration regulates cysteine metabolism to glutathione, sulfate and taurine in rat hepatocytes. J. Nutr. 122, 420–7 (1992). 1542000 10.1093/jn/122.3.420

[b33] Griffith O. W. Biologic and pharmacologic regulation of mammalian glutathione synthesis. Free Radic. Biol. Med. 27, 922–35 (1999). 10569625 10.1016/s0891-5849(99)00176-8

[b34] Linster C. L. & Van Schaftingen E. Vitamin C. Biosynthesis, recycling and degradation in mammals. FEBS J. 274, 1–22 (2007). 10.1111/j.1742-4658.2006.05607.x 17222174

[b35] Meister A. Glutathione-ascorbic acid antioxidant system in animals. J. Biol. Chem. 269, 9397–400 (1994). 8144521

[b36] De Vogel S. . Sarcosine and other metabolites along the choline oxidation pathway in relation to prostate cancer–a large nested case-control study within the JANUS cohort in Norway. Int. J. Cancer 134, 197–206 (2014). 23797698 10.1002/ijc.28347

[b37] Beyer C., Alting I. H. & Backer E. T. Measurement of creatine in urine by creatinase, sarcosine oxidase, and peroxidase reevaluated. Clin. Chem. 39, 1743–4 (1993). 8394792

[b38] McNeil N. I. The contribution of the large intestine to energy supplies in man. Am. J. Clin. Nutr. 39, 338–42 (1984). 6320630 10.1093/ajcn/39.2.338

[b39] Zeisel S. H. & Blusztajn J. K. Choline and human nutrition. Annu. Rev. Nutr. 14, 269–96 (1994). 7946521 10.1146/annurev.nu.14.070194.001413

[b40] Alves S. P. . Does the fat tailed Damara ovine breed have a distinct lipid metabolism leading to a high concentration of branched chain fatty acids in tissues? PLoS ONE 8, e77313 (2013). 24204803 10.1371/journal.pone.0077313 PMC3800059

[b41] Mirzaei-Aghsaghali A. & Maheri-Sis N. Importance of ‘physically effective fibre’ in ruminant nutrition: A review. Ann Biol Res 2, 262–270 (2011).

[b42] Ren M. . Comparing mRNA levels of genes encoding leptin, leptin receptor, and lipoprotein lipase between dairy and beef cattle. Domest Anim Endocrinol 371–381, doi: 10.1016/S0739-7240(02)00179-0 (2002). 12206871

[b43] Eryavuz G., Avci I., Kucukkurt I. & Fidan A. F. Comparison of plasma leptin, insulin and thyroid hormone concentrations and some biochemical parameters between Fat-tailed and Thin-tailed sheep breeds. Rev Med Vet 5, 244–249 (2007).

