# Supplementary material for: The hepatic and skeletal muscle ovine metabolomes as affected by weight loss: a study in three sheep breeds using NMR-metabolomics
Source: Sci Rep. 2016 Dec 14;6:39120. doi: 10.1038/srep39120 (PMC5155430; doi:10.1038/srep39120)
Supplement: Supplementary Material [file srep39120-s1.pdf]

The hepatic and skeletal muscle ovine metabolomes as affected by weight loss: a study in three sheep breeds using NMR-metabolomics

Mariana Palma[1], Tim Scanlon[2], Tanya Kilminster[2], John Milton[3], Chris Oldham[2], Johan Greeff[2], Manolis Matzapetakis[1], André M. Almeida[4,5]

[1]ITQB NOVA, Instituto de Tecnologia Química e Biológica António Xavier, Universidade Nova de Lisboa, Oeiras, Portugal

[2]DAFWA – Department of Agriculture and Food Western Australia, Perth, WA, Australia

[3]UWA – University if Western Australia, Perth, WA, Australia

[4]IBET – Instituto de Biologia Experimental e Tecnológica, Oeiras, Portugal

[5]RUSVM – Ross University School of Veterinary Medicine, Basseterre, St. Kitts and Nevis (West Indies)

Supplementary Figure S1. Multivariate analysis of gastrocnemius muscle (aqueous fraction) metabolites concentration. (A) PCA scores plot for all the experimental groups (NC = 5, PC1 = 38.4 %, PC2 = 8.39 %); (B) PCA scores plot for the three restricted groups (NC = 4, PC1 = 38.2 %, PC2 = 10.5 %); (C) PLS scores plot of Damara growth and restricted groups (NC = 2, R^2 =0.346, Q^2 =0.580, $p_{\text{CV-ANOVA}}$ = 0.0063); (D) Permutation test of the PLS obtained from the Damara growth and restricted groups (100 permutations; Intercepts: R^2 =(0.0, 0.588); Q^2 = (0.0, -0.244)); (E) PLS scores plot of Dorper growth and restricted groups (NC = 3, R^2 =0.602, Q^2 =0.232, $p_{\text{CV-ANOVA}}$ = 1); (F) Permutation test of the PLS obtained from the Dorper growth and restricted groups (100 permutations; Intercepts: R^2 =(0.0, 0.743); Q^2 = (0.0, -0.0635)).

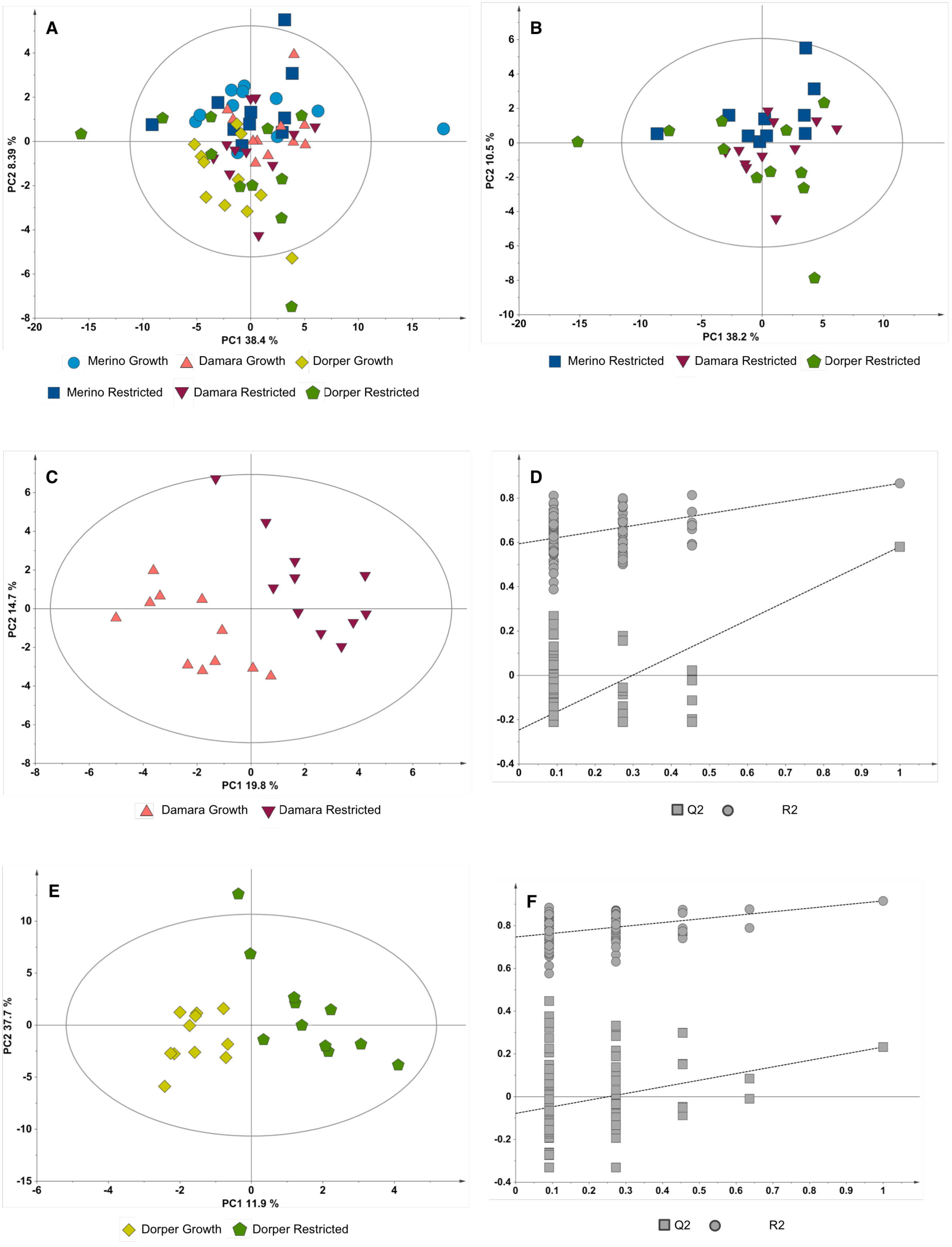

Supplementary Figure S2. Multivariate analysis of gastrocnemius liver (aqueous fraction) metabolites concentration. (A) PLS scores plot of Dorper growth and restricted groups (NC = 3, R^2 =0.697, Q^2 =0.535, $p_{CV\text{-}ANOVA}$ = 0.022); (B) Permutation test of the PLS obtained from the Dorper growth and restricted groups (100 permutations; Intercepts: R^2 =(0.0, 0.661); Q^2 = (0.0, -0.0199)).

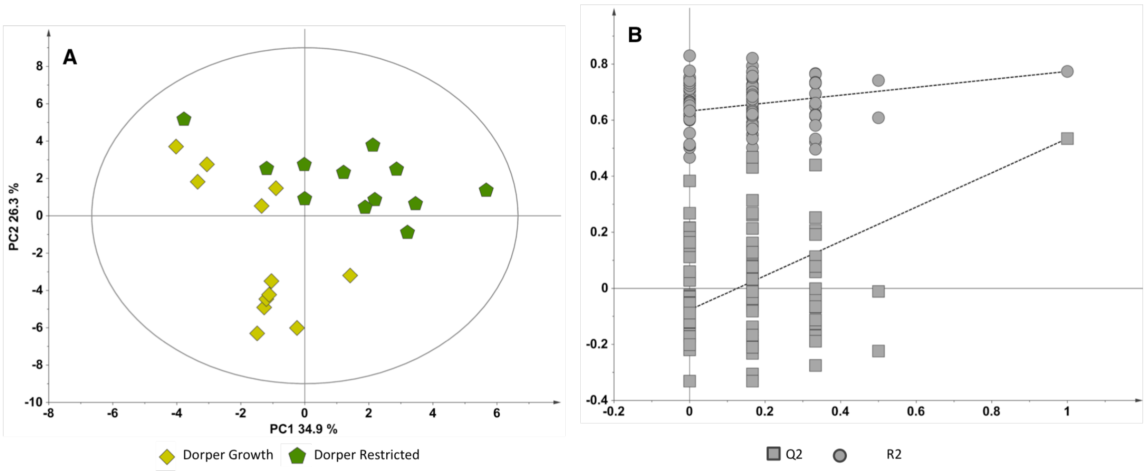

Supplementary Table S1. Identified metabolites in gastrocnemius muscle of Merino, Dorper and Damara sheep breeds (growth and restricted groups). Average concentration (mmol/ g tissue) and standard deviation are shown for each experimental group.

	Merino		Dorper		Damara	
	growth	restricted	growth	restricted	growth	restricted
3-Methylhistidine	2.23E-05 ± 1.82E-05	2.76E-05 ± 2.98E-05	7.08E-06 ± 3.36E-06	6.36E-06 ± 4.28E-06	1.19E-05 ± 3.33E-06	1.49E-05 ± 1.91E-05
β-Alanine	1.21E-04 ± 4.02E-05	1.05E-04 ± 2.58E-05	1.00E-04 ± 5.65E-05	1.06E-04 ± 5.50E-05	1.20E-04 ± 5.31E-05	9.21E-05 ± 3.78E-05
Acetate	8.96E-05 ± 3.04E-05	5.31E-05 ± 2.18E-05	6.04E-05 ± 1.37E-05	6.58E-05 ± 3.16E-05	6.95E-05 ± 1.49E-05	6.98E-05 ± 1.14E-05
Acetyl-L-carnitine[a]	2.57E-03 ± 1.23E-03	4.58E-03* ± 1.94E-03	1.64E-03 ± 5.76E-04	1.59E-03 ± 1.03E-03	2.20E-03 ± 3.79E-04	2.17E-03 ± 7.57E-04
Adenine[a]	8.29E-05 ± 7.53E-05	7.22E-05 ± 4.99E-05	2.08E-05 ± 1.07E-05	5.26E-05* ± 4.11E-05	8.46E-05 ± 4.29E-05	7.35E-05 ± 6.46E-05
Adenosine	7.30E-06 ± 4.27E-06	7.68E-06 ± 1.75E-06	7.27E-06 ± 2.35E-06	5.84E-06 ± 3.65E-06	6.96E-06 ± 1.33E-06	6.63E-06 ± 2.54E-06
ADP/ AMP/ ATP	7.45E-04 ± 5.27E-04	8.81E-04 ± 4.55E-04	1.32E-03 ± 7.84E-04	1.07E-03 ± 9.58E-04	9.43E-04 ± 4.66E-04	1.29E-03 ± 6.91E-04
Alanine	2.36E-03 ± 1.17E-03	2.23E-03 ± 9.02E-04	1.62E-03 ± 7.56E-04	1.80E-03 ± 1.22E-03	3.19E-03 ± 1.12E-03	2.50E-03 ± 1.01E-03
Anserine	5.11E-03 ± 2.49E-03	4.46E-03 ± 2.13E-03	5.48E-03 ± 1.49E-03	5.42E-03 ± 3.08E-03	6.14E-03 ± 1.09E-03	6.66E-03 ± 1.58E-03
Aspartate	1.50E-04 ± 8.45E-05	1.29E-04 ± 6.25E-05	1.22E-04 ± 4.38E-05	1.75E-04 ± 1.23E-04	1.44E-04 ± 4.81E-05	1.61E-04 ± 5.43E-05
Betaine	1.54E-03 ± 5.48E-04	1.61E-03 ± 4.12E-04	8.75E-04 ± 2.80E-04	8.02E-04 ± 5.61E-04	9.88E-04 ± 2.44E-04	1.17E-03 ± 4.31E-04
Carnitine	8.66E-03 ± 4.13E-03	9.19E-03 ± 3.04E-03	4.59E-03 ± 1.82E-03	5.48E-03 ± 3.46E-03	5.98E-03 ± 8.71E-04	6.42E-03 ± 1.16E-03
Carnosine	1.83E-04 ± 3.64E-04	2.59E-04 ± 3.26E-04	4.95E-05 ± 3.02E-05	6.61E-05 ± 4.54E-05	1.32E-04 ± 8.76E-05	9.34E-05 ± 3.81E-05
Choline/ Phosphocholine	8.10E-04 ± 2.81E-04	7.73E-04 ± 2.48E-04	6.42E-04 ± 1.21E-04	7.78E-04 ± 3.33E-04	6.58E-04 ± 1.26E-04	7.56E-04 ± 1.34E-04
Citrate[a]	1.74E-04 ± 8.55E-05	2.69E-04* ± 1.07E-04	1.46E-04 ± 7.50E-05	1.53E-04 ± 1.20E-04	1.88E-04 ± 7.87E-05	2.23E-04 ± 6.82E-05
Creatine/ Creatine phosphate	2.80E-02 ± 1.03E-02	2.80E-02 ± 6.15E-03	2.46E-02 ± 5.06E-03	2.53E-02 ± 1.36E-02	2.59E-02 ± 4.08E-03	2.55E-02 ± 5.13E-03
Creatinine	1.51E-04 ± 7.20E-05	1.28E-04 ± 5.71E-05	1.16E-04 ± 2.99E-05	1.30E-04 ± 8.07E-05	1.56E-04 ± 4.86E-05	1.25E-04 ± 3.30E-05
Formate[a]	5.33E-05 ± 2.03E-05	3.98E-05 ± 7.12E-06	4.82E-05 ± 1.68E-05	3.48E-05* ± 8.20E-06	4.67E-05 ± 1.38E-05	5.58E-05 ± 1.11E-05
Fumarate	4.53E-05 ± 1.85E-05	4.30E-05 ± 1.57E-05	4.34E-05 ± 2.42E-05	3.79E-05 ± 3.05E-05	6.19E-05 ± 1.79E-05	5.75E-05 ± 2.20E-05
GTP	3.53E-05 ± 1.77E-05	3.29E-05 ± 1.05E-05	3.37E-05 ± 1.16E-05	3.57E-05 ± 1.87E-05	3.58E-05 ± 9.98E-06	3.49E-05 ± 9.17E-06
Glucose	1.52E-03 ± 5.24E-04	1.45E-03 ± 4.53E-04	1.22E-03 ± 2.77E-04	1.37E-03 ± 7.61E-04	1.78E-03 ± 3.04E-04	1.67E-03 ± 6.04E-04
Glucose-1-phosphate[a]	6.29E-04 ± 2.65E-04	5.22E-04 ± 2.01E-04	4.97E-04 ± 1.45E-04	4.61E-04 ± 3.15E-04	7.00E-04 ± 2.14E-04	4.80E-04* ± 2.71E-04

Glucose-6-phosphate[a]	2.90E-03 ± 1.21E-03	1.57E-03** ± 7.25E-04	2.69E-03 ± 6.31E-04	1.89E-03 ± 1.20E-03	3.13E-03 ± 1.05E-03	2.24E-03 ± 9.70E-04
Glutamate	4.10E-04 ± 2.26E-04	5.19E-04 ± 1.73E-04	4.80E-04 ± 1.69E-04	6.11E-04 ± 3.28E-04	3.83E-04 ± 9.56E-05	4.83E-04 ± 2.42E-04
Glutamine	4.01E-03 ± 1.85E-03	3.86E-03 ± 2.51E-03	3.53E-03 ± 1.51E-03	4.60E-03 ± 3.03E-03	5.11E-03 ± 1.83E-03	5.17E-03 ± 1.51E-03
Glutathione[a]	1.72E-04 ± 1.03E-04	2.79E-04* ± 1.26E-04	2.20E-04 ± 6.60E-05	2.52E-04 ± 1.29E-04	2.30E-04 ± 9.64E-05	3.13E-04 ± 1.59E-04
Glycerophosphocholine[a]	3.13E-04 ± 1.13E-04	7.71E-05** ± 2.43E-05	3.81E-04 ± 1.89E-04	5.67E-04 ± 2.97E-04	3.98E-04 ± 1.65E-04	5.45E-04 ± 1.82E-04
Glycine[a]	3.48E-03 ± 1.73E-03	1.90E-03* ± 5.35E-04	2.84E-03 ± 7.71E-04	1.87E-03* ± 1.31E-03	2.24E-03 ± 4.39E-04	1.76E-03 ± 7.04E-04
Histidine	7.53E-06 ± 2.96E-06	6.02E-06 ± 2.40E-06	3.06E-06 ± 1.03E-06	3.77E-06 ± 2.33E-06	5.40E-06 ± 2.01E-06	4.20E-06 ± 1.96E-06
Homocysteine	1.47E-03 ± 8.12E-04	1.36E-03 ± 5.47E-04	1.22E-03 ± 4.30E-04	1.41E-03 ± 6.20E-04	1.29E-03 ± 3.50E-04	1.41E-03 ± 4.36E-04
IMP[a]	2.46E-03 ± 1.05E-03	1.89E-03 ± 7.44E-04	1.46E-03 ± 6.49E-04	1.70E-03 ± 8.59E-04	2.45E-03 ± 7.02E-04	1.72E-03* ± 7.69E-04
Inosine	1.78E-04 ± 8.73E-05	1.27E-04 ± 5.61E-05	9.23E-05 ± 4.47E-05	9.90E-05 ± 7.30E-05	1.59E-04 ± 6.48E-05	1.39E-04 ± 8.86E-05
Isoleucine[a]	9.29E-05 ± 3.88E-05	7.76E-05 ± 2.02E-05	8.58E-05 ± 2.36E-05	8.18E-05 ± 4.78E-05	1.09E-04 ± 1.35E-05	9.05E-05* ± 2.47E-05
Lactate	4.50E-02 ± 1.83E-02	4.12E-02 ± 1.11E-02	3.87E-02 ± 3.96E-03	4.12E-02 ± 1.55E-02	4.92E-02 ± 7.93E-03	4.43E-02 ± 1.02E-02
Leucine[a]	1.40E-04 ± 5.55E-05	1.12E-04 ± 2.55E-05	1.27E-04 ± 3.67E-05	1.17E-04 ± 5.31E-05	1.64E-04 ± 2.20E-05	1.34E-04* ± 3.11E-05
Malonate	5.48E-03 ± 2.14E-03	5.72E-03 ± 2.33E-03	2.93E-03 ± 1.14E-03	3.75E-03 ± 1.71E-03	5.11E-03 ± 1.68E-03	5.85E-03 ± 1.90E-03
Methylmalonate	3.02E-04 ± 1.67E-04	2.70E-04 ± 6.69E-05	2.57E-04 ± 2.62E-05	2.68E-04 ± 9.76E-05	3.16E-04 ± 4.53E-05	2.85E-04 ± 6.91E-05
Myo-Inositol	1.21E-03 ± 7.57E-04	1.69E-03 ± 6.59E-04	1.25E-03 ± 7.87E-04	1.58E-03 ± 9.12E-04	1.15E-03 ± 7.34E-04	1.29E-03 ± 8.69E-04
NAD^+/ $NADP^+$	1.16E-04 ± 6.46E-05	8.57E-05 ± 2.90E-05	1.27E-04 ± 5.27E-05	1.00E-04 ± 5.21E-05	1.19E-04 ± 3.17E-05	1.14E-04 ± 5.84E-05
Nicotinurate	1.44E-04 ± 5.72E-05	1.26E-04 ± 2.96E-05	1.00E-04 ± 3.06E-05	1.16E-04 ± 4.09E-05	1.42E-04 ± 3.98E-05	1.19E-04 ± 3.22E-05
Phenylalanine[a]	4.90E-05 ± 1.62E-05	3.86E-05 ± 1.07E-05	4.39E-05 ± 9.47E-06	3.46E-05 ± 1.34E-05	5.34E-05 ± 8.22E-06	3.97E-05** ± 8.62E-06
Proline	2.42E-04 ± 1.08E-04	2.73E-04 ± 9.73E-05	2.62E-04 ± 8.01E-05	3.32E-04 ± 1.78E-04	2.52E-04 ± 5.58E-05	3.24E-04 ± 1.68E-04
Pyruvate	2.04E-04 ± 1.13E-04	2.10E-04 ± 1.43E-04	1.65E-04 ± 5.75E-05	1.97E-04 ± 9.99E-05	2.70E-04 ± 7.93E-05	2.37E-04 ± 1.08E-04
Riboflavin	1.42E-05 ± 4.41E-06	1.28E-05 ± 5.83E-06	1.71E-05 ± 5.87E-06	1.93E-05 ± 9.60E-06	1.59E-05 ± 3.76E-06	1.60E-05 ± 6.08E-06
Succinate	1.01E-03 ± 3.87E-04	1.01E-03 ± 3.72E-04	6.67E-04 ± 3.30E-04	6.43E-04 ± 3.80E-04	1.19E-03 ± 4.14E-04	1.08E-03 ± 3.87E-04
Taurine[a]	5.35E-03 ± 3.30E-03	2.96E-03* ± 1.13E-03	6.32E-03 ± 2.82E-03	3.88E-03* ± 2.35E-03	1.44E-02 ± 4.40E-03	8.77E-03** ± 2.02E-03
Tyrosine[a]	5.50E-05 ± 2.65E-05	3.52E-05* ± 1.34E-05	5.04E-05 ± 1.68E-05	3.83E-05 ± 1.55E-05	6.26E-05 ± 1.60E-05	4.59E-05* ± 1.17E-05

UDP-glucose	3.22E-05 ± 2.13E-05	3.14E-05 ± 1.11E-05	4.02E-05 ± 1.20E-05	3.47E-05 ± 2.26E-05	3.66E-05 ± 1.10E-05	3.59E-05 ± 2.15E-05
Uridine	1.94E-05 ± 6.83E-06	1.54E-05 ± 8.41E-06	1.53E-05 ± 5.28E-06	1.15E-05 ± 6.99E-06	1.89E-05 ± 4.49E-06	1.48E-05 ± 5.91E-06
Valine[a]	2.13E-04 ± 9.95E-05	1.48E-04 ± 3.51E-05	1.95E-04 ± 5.68E-05	1.86E-04 ± 1.07E-04	2.25E-04 ± 3.97E-05	1.87E-04* ± 3.74E-05
Xanthine	5.83E-05 ± 5.44E-05	2.63E-05 ± 2.33E-05	1.85E-05 ± 6.97E-06	2.59E-05 ± 3.26E-05	1.56E-05 ± 5.50E-06	3.66E-05 ± 5.63E-05

Key: (a) metabolites with significant differences between growth and restricted groups in at least one breed; (*) $p < 0.05$ when compared with growth groups of the same breed; (**) $p < 0.01$ when compared with growth groups of the same breed.

Supplementary Table S2. PCA loadings values of principal component 1 (p[1]) and principal component 2 (p[2]) of muscle from Merino growth and restricted groups (see scores in Figure 2B). Highlighted values are the more representatives for the group clustering.

metabolite	p[1]	p[2]
choline/phosphocholine	0,0934444	-0,00916684
lactate	0,187801	-0,0257329
riboflavin	0,108283	-0,0279286
homocysteine	0,13865	-0,0281437
GTP	0,161932	-0,0284619
adenine	0,153523	-0,0293082
adenosine	0,0910446	-0,0313269
isoleucine	0,17947	-0,0335238
methylmalonate	0,187916	-0,0373872
histidine	0,145532	-0,0400434
glucose	0,16066	-0,0483827
leucine	0,176612	-0,0493796
glucose-1-phosphate	0,164189	-0,0517669
beta-alanine	0,140296	-0,0566436
phenylalanine	0,176972	-0,0739192
IMP	0,162193	-0,0880994
tyrosine	0,163835	-0,0916036
inosine	0,13526	-0,0961437
creatine	0,0984559	-0,112648
valine	0,171368	-0,116997
taurine	0,160134	-0,160068
anserine	0,0872286	-0,170245
NAD+/NADP+	0,14135	-0,187977
formate	0,128394	-0,189047
glycine	0,143278	-0,19155
xanthine	-0,015431	-0,20734
glucose-6-phosphate	0,123557	-0,305008
creatine/creatine phosphate	0,18423	0,00272127
nicotinurate	0,174855	0,0223421
aspartate	0,167016	0,0305508
UDP-glucose	0,160648	0,0329277
acetate	0,158195	0,0358494
alanine	0,170251	0,036475
uridine	0,133736	0,0596227
pyruvate	0,106272	0,0650886
carnitine	0,169322	0,0885765
fumarate	0,170695	0,0933712
ADP/AMP/ATP	0,135175	0,106753
proline	0,15098	0,110592
succinate	0,156269	0,119165
betaine	0,048716	0,122313
malonate	0,150058	0,131919
3-methylhistidine	0,0158208	0,136634
carnosine	0,00697562	0,164795
glutamate	0,157805	0,179145
glutamine	0,0984383	0,184886
glutathione	0,0993436	0,244599
acetyl-L-carnitine	0,080261	0,261162
myo-inositol	0,109134	0,261164
citrate	0,118022	0,31952
glycerophosphocholine	0,020397	0,338363

Supplementary Table S3. Identified metabolites in liver of Merino, Dorper and Damara sheep breeds (growth and restricted groups). Average concentration (mmol/ g tissue) and standard deviation are shown for each experimental group.

	Merino		Dorper		Damara	
	growth	restricted	growth	restricted	growth	restricted
3-Hydroxybutyrate[a]	3.30E-04 ± 1.00E-04	4.67E-04* ± 1.40E-04	2.35E-04 ± 9.97E-05	3.18E-04 ± 1.40E-04	2.91E-04 ± 7.93E-05	2.67E-04 ± 1.38E-04
Methylhistidine	1.30E-04 ± 1.39E-04	1.05E-04 ± 1.01E-04	1.85E-04 ± 1.88E-04	1.01E-04 ± 1.03E-04	5.79E-05 ± 6.53E-05	6.70E-05 ± 7.89E-05
ADP AMP ATP	8.00E-04 ± 3.30E-04	9.49E-04 ± 3.27E-04	7.20E-04 ± 3.51E-04	7.56E-04 ± 2.87E-04	9.19E-04 ± 1.74E-04	6.90E-04 ± 3.80E-04
Acetate[a]	5.89E-04 ± 1.26E-04	8.29E-04** ± 1.72E-04	4.94E-04 ± 2.32E-04	5.77E-04 ± 2.47E-04	6.08E-04 ± 1.31E-04	4.32E-04* ± 2.31E-04
Adenine[a]	3.30E-04 ± 1.46E-04	3.03E-04 ± 1.63E-04	1.64E-04 ± 9.73E-05	1.87E-04 ± 1.02E-04	3.03E-04 ± 4.54E-05	1.63E-04** ± 1.19E-04
Adenosylhomocysteine	4.41E-05 ± 2.52E-05	5.45E-05 ± 2.36E-05	2.67E-05 ± 2.02E-05	4.36E-05 ± 2.24E-05	4.75E-05 ± 1.94E-05	4.17E-05 ± 2.20E-05
Alanine[a]	1.97E-03 ± 4.86E-04	1.38E-03** ± 4.18E-04	1.78E-03 ± 8.76E-04	1.37E-03 ± 4.57E-04	2.38E-03 ± 2.77E-04	1.53E-03** ± 7.19E-04
Ascorbate[a]	1.24E-03 ± 3.70E-04	7.67E-04** ± 3.53E-04	9.23E-04 ± 4.95E-04	7.21E-04 ± 3.23E-04	1.15E-03 ± 2.52E-04	8.22E-04* ± 4.12E-04
Aspartate	8.23E-04 ± 1.34E-04	9.19E-04 ± 3.37E-04	7.53E-04 ± 3.23E-04	9.52E-04 ± 3.94E-04	1.17E-03 ± 2.95E-04	9.09E-04 ± 4.43E-04
Benzoate	2.37E-05 ± 1.65E-05	2.30E-05 ± 1.53E-05	1.35E-05 ± 1.59E-05	1.21E-05 ± 9.44E-06	2.67E-05 ± 1.46E-05	1.93E-05 ± 8.36E-06
Betaine	4.22E-04 ± 8.76E-05	4.73E-04 ± 8.99E-05	3.07E-04 ± 2.03E-04	4.22E-04 ± 1.97E-04	3.55E-04 ± 1.54E-04	3.18E-04 ± 1.84E-04
Carnitine[a]	5.68E-04 ± 3.16E-04	5.77E-04 ± 3.47E-04	6.40E-04 ± 3.45E-04	9.74E-04* ± 4.27E-04	7.03E-04 ± 2.61E-04	7.08E-04 ± 3.79E-04
Carnosine	3.24E-05 ± 2.48E-05	4.80E-05 ± 1.72E-05	4.11E-05 ± 2.34E-05	3.57E-05 ± 3.56E-05	5.20E-05 ± 6.45E-05	2.74E-05 ± 1.92E-05
Choline/ Acetylcholine/ Phosphocholine[a]	5.97E-04 ± 3.04E-04	8.77E-04 ± 6.55E-04	4.55E-04 ± 3.61E-04	5.39E-04 ± 2.70E-04	6.21E-04 ± 2.58E-04	3.43E-04* ± 2.27E-04
Citrate[a]	3.64E-04 ± 1.22E-04	2.57E-04 ± 1.34E-04	3.23E-04 ± 1.36E-04	2.49E-04 ± 1.08E-04	4.87E-04 ± 1.21E-04	2.92E-04** ± 1.83E-04
Creatine/ Creatine phosphate[a]	1.66E-03 ± 5.20E-04	2.60E-03** ± 7.24E-04	1.47E-03 ± 6.77E-04	1.76E-03 ± 8.34E-04	1.59E-03 ± 3.28E-04	1.68E-03 ± 1.04E-03
Creatinine	2.62E-04 ± 2.11E-04	2.59E-04 ± 5.52E-05	2.33E-04 ± 1.61E-04	2.33E-04 ± 1.35E-04	2.24E-04 ± 6.99E-05	2.12E-04 ± 1.23E-04
Formate[a]	1.21E-04 ± 7.59E-05	1.34E-04 ± 5.55E-05	3.89E-05 ± 3.40E-05	4.99E-05 ± 5.04E-05	1.21E-04 ± 5.60E-05	4.80E-05** ± 2.52E-05
Fumarate	1.07E-04 ± 4.85E-05	1.26E-04 ± 6.44E-05	8.43E-05 ± 3.99E-05	1.20E-04 ± 5.73E-05	1.06E-04 ± 3.06E-05	1.11E-04 ± 7.94E-05
Glucose	8.68E-02 ± 1.81E-02	7.76E-02 ± 1.53E-02	8.03E-02 ± 3.70E-02	7.12E-02 ± 2.63E-02	9.10E-02 ± 1.51E-02	7.71E-02 ± 3.11E-02
Glutamate	3.98E-03 ± 9.35E-04	3.38E-03 ± 1.20E-03	4.16E-03 ± 1.94E-03	3.90E-03 ± 1.73E-03	3.87E-03 ± 8.97E-04	3.74E-03 ± 1.77E-03
Glutathione[a]	2.53E-03 ± 7.38E-04	1.20E-03** ± 4.27E-04	2.24E-03 ± 1.36E-03	1.51E-03 ± 6.41E-04	2.54E-03 ± 4.75E-04	1.93E-03 ± 1.26E-03

Glycerophosphocholine	1.15E-02 ± 1.48E-03	1.15E-02 ± 2.13E-03	7.36E-03 ± 2.92E-03	8.79E-03 ± 3.12E-03	9.40E-03 ± 1.00E-03	8.40E-03 ± 2.85E-03
Glycine	2.98E-03 ± 2.28E-03	5.04E-03 ± 3.07E-03	2.95E-03 ± 2.62E-03	3.52E-03 ± 2.60E-03	2.51E-03 ± 2.20E-03	3.23E-03 ± 2.13E-03
Histamine	7.22E-05 ± 4.98E-05	7.17E-05 ± 5.11E-05	8.66E-05 ± 1.28E-04	5.94E-05 ± 6.40E-05	7.67E-05 1.05E-04	4.48E-05 ± 6.62E-05
Histidine	1.76E-05 ± 1.77E-05	3.90E-05 ± 6.67E-05	4.60E-05 ± 6.54E-05	6.57E-05 ± 8.10E-05	4.27E-05 ± 4.42E-05	2.96E-05 ± 2.65E-05
Inosine	1.38E-03 ± 3.39E-04	1.15E-03 ± 5.63E-04	1.04E-03 ± 5.13E-04	1.16E-03 ± 4.13E-04	1.15E-03 ± 1.61E-04	1.00E-03 ± 4.30E-04
Isoleucine	2.16E-04 ± 3.05E-05	2.35E-04 ± 4.58E-05	1.97E-04 ± 9.67E-05	2.05E-04 ± 7.34E-05	2.17E-04 ± 3.94E-05	2.01E-04 ± 9.25E-05
Lactate[a]	2.33E-02 ± 4.36E-03	1.97E-02* ± 2.99E-03	1.65E-02 ± 7.78E-03	1.50E-02 ± 5.30E-03	2.10E-02 ± 2.70E-03	1.48E-02** ± 5.34E-03
Leucine	5.15E-04 ± 1.33E-04	5.56E-04 ± 1.40E-04	3.98E-04 ± 1.92E-04	4.63E-04 ± 1.47E-04	5.15E-04 ± 7.97E-05	4.53E-04 ± 1.86E-04
Lysine	1.84E-04 ± 8.02E-05	2.01E-04 ± 8.48E-05	2.27E-04 ± 9.18E-05	2.33E-04 ± 1.03E-04	2.77E-04 ± 7.91E-05	2.30E-04 ± 1.05E-04
myo-Inositol	1.79E-03 ± 8.03E-04	2.39E-03 ± 6.31E-04	1.50E-03 ± 6.14E-04	1.57E-03 ± 5.63E-04	1.61E-03 ± 6.65E-04	1.34E-03 ± 6.57E-04
NAD^+/ $NADP^+$/ NADPH	3.39E-04 ± 7.57E-05	3.27E-04 ± 8.69E-05	2.22E-04 ± 1.10E-04	2.43E-04 ± 9.46E-05	3.08E-04 ± 3.12E-05	2.58E-04 ± 1.28E-04
Nicotinurate	4.05E-04 ± 1.20E-04	3.85E-04 ± 2.04E-04	3.64E-04 ± 2.19E-04	3.14E-04 ± 1.42E-04	3.37E-04 ± 6.96E-05	2.70E-04 ± 1.15E-04
Phenylalanine	9.77E-05 ± 2.70E-05	9.26E-05 ± 1.97E-05	7.96E-05 ± 3.84E-05	8.75E-05 ± 3.51E-05	8.81E-05 ± 2.65E-05	8.19E-05 ± 3.41E-05
Pyruvate	1.01E-04 ± 5.32E-05	1.22E-04 ± 6.77E-05	8.44E-05 ± 7.04E-05	1.09E-04 ± 7.39E-05	1.02E-04 ± 7.93E-05	6.27E-05 ± 5.67E-05
Sarcosine[a]	2.50E-05 ± 1.06E-05	6.57E-05* ± 4.68E-05	1.91E-05 ± 9.89E-06	4.99E-05** ± 3.33E-05	2.54E-05 ± 9.92E-06	2.84E-05 ± 1.94E-05
Succinate[a]	1.94E-03 ± 7.03E-04	1.10E-03* ± 7.40E-04	1.40E-03 ± 9.42E-04	1.01E-03 ± 5.22E-04	1.58E-03 ± 6.35E-04	1.35E-03 ± 6.22E-04
Taurine	3.14E-03 ± 1.43E-03	2.90E-04 ± 5.48E-04	2.11E-03 ± 6.67E-04	2.05E-03 ± 1.33E-03	2.25E-03 ± 1.36E-03	2.46E-03 ± 1.13E-03
Tyrosine	1.06E-04 ± 2.14E-05	1.13E-04 ± 1.97E-05	9.53E-05 ± 4.17E-05	1.07E-04 ± 3.94E-05	1.18E-04 ± 1.97E-05	9.84E-05 ± 4.55E-05
UDP-glucose/ UDP-glucoronate[a]	2.37E-04 ± 6.03E-05	1.69E-04* ± 6.06E-05	1.79E-04 ± 8.35E-05	1.99E-04 ± 8.58E-05	1.82E-04 ± 4.43E-05	1.35E-04* ± 5.16E-05
UMP	2.16E-04 ± 6.39E-05	1.99E-04 ± 9.61E-05	1.73E-04 ± 1.20E-04	2.06E-04 ± 1.06E-04	2.37E-04 ± 6.70E-05	1.91E-04 ± 1.19E-04
Uridine	2.00E-04 ± 8.53E-05	1.89E-04 ± 7.52E-05	1.73E-04 ± 9.29E-05	2.16E-04 ± 9.06E-05	2.52E-04 ± 4.53E-05	1.79E-04 ± 1.47E-04
Valine	2.85E-04 ± 4.72E-05	2.68E-04 ± 5.60E-05	2.89E-04 ± 1.49E-04	3.24E-04 ± 1.10E-04	2.84E-04 ± 7.18E-05	2.92E-04 ± 1.45E-04
Xanthine	9.03E-05 ± 1.28E-04	5.88E-05 ± 6.43E-05	1.07E-04 ± 2.08E-04	1.94E-04 ± 2.51E-04	1.51E-04 ± 1.73E-04	1.10E-04 ± 2.10E-04
Xanthosine	5.53E-05 ± 1.95E-05	6.54E-05 ± 1.56E-05	3.13E-05 ± 1.46E-05	4.47E-05 ± 1.85E-05	3.99E-05 ± 9.11E-06	3.73E-05 ± 1.57E-05

Key: (a) metabolites with significant differences between growth and restricted groups in at least one breed; (*) $p < 0.05$ when compared with growth groups of the same breed; (**) $p < 0.01$ when compared with growth groups of the same breed.

Supplementary Table S4. PCA loadings values of principal component 1 (p[1]) and principal component 2 (p[2]) of liver from all experimental groups (see scores in Figure 4A). Highlighted values are the more representatives for the group clustering.

metabolite	p[1]	p[2]
lysine	0,127616	-0,00836563
tyrosine	0,217153	-0,0101978
leucine	0,213215	-0,0182982
fumarate	0,16816	-0,0194202
isoleucine	0,218857	-0,0270153
valine	0,193653	-0,0282933
myo-inositol	0,126898	-0,0344818
aspartate	0,175622	-0,0405529
betaine	0,182334	-0,0429109
carnitine	0,0864109	-0,0503634
glycerophosphocholine	0,199769	-0,0594424
3-hydroxybutyrate	0,17255	-0,0637507
phenylalanine	0,195149	-0,0656461
creatine/creatine phosphate	0,150869	-0,0656852
sarcosine	0,0993925	-0,117427
NAD+/NADP+/NADPH	0,15416	-0,123242
acetate	0,181122	-0,124148
histamine	0,0689646	-0,162512
taurine	0,0889907	-0,165381
xanthosine	0,13541	-0,182926
carnosine	0,0751018	-0,186254
formate	0,11196	-0,254873
pyruvate	0,10112	-0,257031
adenosylhomocysteine	0,0905311	-0,265901
benzoate	0,0699395	-0,303525
glycine	0,0717862	-0,344065
choline/acetylcholine/phosphocholine	0,126998	0,00421237
histidine	0,0423974	0,0065039
ADP/AMP/ATP	0,160462	0,0274156
methylhistidine	0,0542767	0,0421994
glutamate	0,185099	0,0518193
adenine	0,13848	0,0601666
UDP-glucose/UDP-glucoronate	0,153788	0,0665798
uridine	0,154128	0,0822441
UMP	0,142359	0,0970162
creatine	0,109538	0,105426
ascorbate	0,136358	0,118421
xanthine	0,0749608	0,134583
nicotinurate	0,163784	0,140839
lactate	0,196794	0,147192
inosine	0,177502	0,154198
citrate	0,133787	0,158362
glucose	0,18902	0,164965
alanine	0,171358	0,208244
glutathione	0,143614	0,219198
succinate	0,102549	0,337444

Supplementary Table S5. PCA loadings values of principal component 1 (p[1]) and principal component 2 (p[2]) of liver from the three restricted groups (see scores in Figure 4B). Highlighted values are the more representatives for the group clustering.

metabolite	p[1]	p[2]
isoleucine	0,208316	-0,000775746
phenylalanine	0,188558	-0,00329607
nocotinurate	0,143321	-0,00548825
lactate	0,190172	-0,00862534
creatine/creatine phosphate	0,170103	-0,0123637
UDP-glucose/UDP-glucoronate	0,14332	-0,0217326
tyrosine	0,206108	-0,0388418
lysine	0,13228	-0,0388863
creatine	0,138003	-0,0522449
histidine	0,0606518	-0,0612253
aspartate	0,17443	-0,071565
glutamate	0,17281	-0,0871191
ascorbate	0,15292	-0,0905637
citrate	0,158871	-0,103218
fumarate	0,161439	-0,107697
valine	0,185765	-0,115555
UMP	0,148757	-0,121438
carnitine	0,116598	-0,131282
glutathione	0,142582	-0,154829
glucose	0,18041	-0,167011
uridine	0,145556	-0,174022
inosine	0,146805	-0,18577
alanine	0,168406	-0,205698
xanthine	0,0942055	-0,238436
succinate	0,0769011	-0,279004
ADP/AMP/ATP	0,180266	0,00599214
leucine	0,198651	0,0248222
NAD+/NADP+/NADPH	0,158748	0,028534
glycerophosphocholine	0,18985	0,060954
betaine	0,191049	0,0765777
adenine	0,121361	0,0798769
myo-inositol	0,136747	0,0845144
methylhistidine	0,046819	0,0851829
sarcosine	0,120288	0,12591
choline/acetylcholine/phosphocholine	0,117606	0,126589
3-hydroxybutyrate	0,18354	0,131976
histamine	0,0530351	0,145616
acetate	0,172174	0,152669
carnosine	0,12277	0,184341
xanthosine	0,15219	0,207178
taurine	0,0854165	0,228516
pyruvate	0,0859815	0,234773
benzoate	0,0707271	0,255549
glycine	0,0886229	0,263101
adenosylhomocysteine	0,0886352	0,274663
formate	0,121623	0,296229

Supplementary Table S6. PCA loadings values of principal component 1 (p[1]) and principal component 2 (p[2]) of liver from Merino control and restricted groups (see scores in Figure 4C). Highlighted values are the more representatives for the group clustering.

metabolite	p[1]	p[2]
xanthine	-0,130986	-0,0215009
phenylalanine	0,227905	-0,0283117
UMP	0,127628	-0,0521813
uridine	0,0977984	-0,0590317
adenine	0,0628757	-0,0592536
methylhistidine	0,141309	-0,0787875
glutamate	0,153095	-0,097344
nicotinurate	0,251954	-0,0973478
ascorbate	-0,0910994	-0,0998218
UDP-glucose/UDP-glucoronate	0,038097	-0,100275
citrate	-0,00360237	-0,144997
creatine	0,125737	-0,155774
glutathione	-0,00830276	-0,225313
inosine	0,051935	-0,228885
alanine	0,119323	-0,257885
glucose	0,0754439	-0,273554
lactate	0,166109	-0,287578
succinate	0,075375	-0,320218
fumarate	0,168831	0,00107662
lysine	-0,107692	0,00221375
glycerophosphocholine	0,193145	0,0142183
valine	0,233315	0,0247463
NAD+/NADP+/NADPH	-0,065732	0,0299473
leucine	0,256368	0,0316425
tyrosine	0,284198	0,039383
choline/acetylcholine/phosphocholine	0,195512	0,0450083
taurine	0,0405945	0,0504528
betaine	0,288543	0,0543983
histidine	0,0189755	0,0555584
aspartate	0,200786	0,0787987
isoleucine	0,308628	0,0831473
3-hydroxybutyrate	0,224642	0,0971955
ADP/AMP/ATP	0,0458819	0,115534
xanthosine	0,118581	0,119332
pyruvate	0,0877144	0,125332
carnitine	-0,164151	0,131304
sarcosine	0,179643	0,135232
histamine	0,0375752	0,138799
creatine/creatine phosphate	-0,0620337	0,16219
carnosine	0,0048895	0,169906
adenosylhomocysteine	0,0725635	0,171096
myo-inositol	0,0228209	0,180632
acetate	0,140317	0,201524
benzoate	-0,00294643	0,211504
formate	0,0243885	0,227965
glycine	0,077779	0,280858

Supplementary Table S7. PCA loadings values of principal component 1 (p[1]) and principal component 2 (p[2]) of liver from Damara growth and restricted groups (see scores in Figure 4D). Highlighted values are the more representatives for the group clustering.

metabolite	p[1]	p[2]
glycine	0,0244041	0,330112
methylhistidine	0,0395212	0,142924
adenosylhomocysteine	0,049878	0,225319
carnosine	0,0571769	0,139833
histamine	0,0649823	0,352769
pyruvate	0,069471	0,29267
taurine	0,0777508	0,187248
histidine	0,0854182	-0,0733942
sarcosine	0,089201	0,0101228
benzoate	0,0895776	0,308548
succinate	0,114866	-0,277505
choline/acetylcholine/phosphocholine	0,117652	-0,172304
myo-inositol	0,117797	-0,073074
formate	0,120196	0,0919685
betaine	0,121406	-0,0799325
creatine	0,125324	-0,0839733
nicotinurate	0,126424	-0,147877
xanthine	0,127075	-0,07777
carnitine	0,138737	-0,0065273
lysine	0,146226	0,151516
xanthosine	0,146916	0,0853477
UDP-glucose/UDP-glucoronate	0,147759	-0,160506
UMP	0,154387	-0,0736292
3-hydroxybutyrate	0,15474	-0,105817
adenine	0,161653	-0,167643
phenylalanine	0,162582	0,154978
fumarate	0,170056	0,0778041
aspartate	0,170086	0,10321
uridine	0,171311	-0,0805676
ADP/AMP/ATP	0,172519	-0,202394
acetate	0,174108	-0,072834
creatine/creatine phosphate	0,174122	-0,0683038
citrate	0,174285	-0,0359612
glutathione	0,175249	-0,0786109
valine	0,175946	0,123836
glutamate	0,177562	0,15096
lactate	0,178027	-0,11048
alanine	0,178856	-0,0699502
ascorbate	0,179167	-0,071083
NAD+/NADP+/NADPH	0,182011	0,106688
glycerophosphocholine	0,186098	0,0281151
inosine	0,190939	-0,00196768
leucine	0,191633	0,00893044
tyrosine	0,192082	0,0391107
isoleucine	0,192687	0,0726498
glucose	0,198681	0,082695

Supplementary Table S8. Summary of previous results of the same animals.

Live weight variation (%) between day 0 and day 42: (-) decrease, (+) increase, significant differences ($p<0.05$) were found for all experimental groups in a comparison of day 42 and day 0, and between the two groups of the same breed at day 42.

Carcass weights (kg) and yields (%): Data shown as mean ± standard deviation. Different letters (a,b) in each row for each nutritional treatment indicate significant differences ($p<0.05$) between breeds. Different letters (A, B, C) indicate significant differences ($p<0.05$) between nutritional treatments for the same breed: A – Merino, B – Damara, C – Dorper.

Liver weight (g): Data shown as mean ± standard deviation. No significant differences ($p<0.05$) were observed between breeds. Star (*) indicates significant differences between nutritional treatments ($p<0.05$).

Meat characteristics (colour, pH, eye muscle/longissimus dorsi muscle dimensions): Data shown as mean ± standard deviation. Different letters (a, b, c) in each row indicate significant differences ($p<0.05$) between breeds, for each parameter (colour and dimensions). Star (*) indicates significant differences between nutritional treatments ($p<0.05$). Colour (redness, yellowness and lightness), higher values means redder/yellower/lighter.

Plasma concentrations of leptin and insulin (µU/ml): Data shown as means ± standard error. No interaction effect of breed and feed restriction was observed. Different letters (a, b) in each row indicate significant differences. Asterisks: (*) indicates significant differences ($p<0.05$), (**) indicates significant differences ($p<0.001$). Leptin: significant differences ($p<0.001$) were observed between feed restriction and growth groups between day 0 and day 42 in all groups.

Adapted from: Almeida et al. (2013) Trop Anim Health Prod. 45:1305-1311 (Reference No. 7) and Almeida et al. (2016) PlosOne. DOI:10.1371 (Reference No. 13). Shown here for contextual reasons only.

		Merino		Dorper		Damara	
		Growth	Restricted	Growth	Restricted	Growth	Restricted
**Live weight variation (%)**		+10	-14	+13	-12	+7	-12
**Carcass hot weight (kg)**		14.81±2.18 a	11.11±1.10 aA	20.17±1.55 b	14.90±1.93 bC	20.27±3.61 b	16.70±3.06 bB
**Carcass yield (%)**		39.11±1.55 a	38.02±2.62 a	44.53±2.66 b	43.30±2.66 b	43.89±3.17 b	44.64±3.17 b
**Liver weight (g)**		612.85±76.54 *	375.40±40.98	696.34±94.00 *	419.33±95.85	637.69±101.80 *	470.45±61.57
**Colour**	**redness**	14.39±0.78 a*	16.28±1.03 a	14.50±1.54 a	15.31±1.05 ab	13.88±0.93 a	14.11±1.08 b
	**yellowness**	6.15±0.88 a	6.06±1.02 a	5.97±0.90 a	5.84±0.75 a	5.64±1.37 a	5.57±0.60 a
	**lightness**	42.35±2.47 a	41.26±1.12 a	40.00±1.41 b	40.78±1.89 a	38.07±1.76 b	37.82±1.74 b
**pH**		5.79±0.06 a*	5.92±0.61 a	5.89±0.06 b	5.96±0.08 a	5.78±0.05 a	5.83±0.04 b
**Eye muscle**	**area (cm^2)**	1065.33±211.90 a	800.87±159.89 a	1569.31±286.45 b*	1177.07±186.60 b	1485.96±241.86 b	1373.33±246.73 b
	**diameter (mm)**	23.08±3.37 a*	18.71±3.25 a	30.18±4.24 b*	24.75±2.67 b	29.91±3.97 b	27.83±2.89 b
	**width (mm)**	57.33±4.23 a	53.27±3.28 a	64.64±3.63 b*	59.17±3.81 b	62.00±3.74 ab	61.33±5.07 b
**Leptin (μU/ml)**	**Day 0**	0.501±0.015	0.541±0.018 a**	0.547±0.032	0.533±0.023 a**	0.773±0.084	0.810±0.088 b**
	**Day 42**	0.883±0.057	0.638±0.049 a*	0.951±0.055	0.627±0.048 a*	1.084±0.126	0.873±0.104 b*
**Insulin (μU/ml)**	**Day 0**	3.671±0.469	2.703±0.271 a**	3.463±0.580	2.815±0.278 a**	7.632±1.259	5.828±0.981 b**
	**Day 42**	4.223±0.470	2.322±0.232 a**	4.318±0.348	2.067±0.11 a**	6.938±0.821	3.958±0.468 b**
